# Development of a europium nanoparticles lateral flow immunoassay for NGAL detection in urine and diagnosis of acute kidney injury

**DOI:** 10.1186/s12882-021-02493-w

**Published:** 2022-01-14

**Authors:** Moli Yin, Yuanwang Nie, Hao Liu, Lei Liu, Lu Tang, Yuan Dong, Chuanmin Hu, Huiyan Wang

**Affiliations:** 1 Jilin Collaborative Innovation Center for Antibody Engineering, Jilin Medical University, 132013 Jilin, PR China; 2 Academy of laboratory, Jilin Medical University, 132013 Jilin, PR China

**Keywords:** neutrophil gelatinase-associated lipocalin (NGAL), monoclonal antibody, lateral flow immunoassay, acute kidney injury (AKI)

## Abstract

**Background:**

AKI is related to severe adverse outcomes and mortality with Coronavirus Disease 2019 (COVID-19) patients, that early diagnosed and intervened is imperative. Neutrophil gelatinase-associated lipocalin (NGAL) is one of the most promising biomarkers for detection of acute kidney injury (AKI), but current detection methods are inadequacy, so more rapid, convenient and accuracy methods are needed to detect NGAL for early diagnosis of AKI. Herein, we established a rapid, reliable and accuracy lateral flow immunoassay (LFIA) based on europium nanoparticles (EU-NPS) for the detection of NGAL in human urine specimens.

**Methods:**

A double-antibody sandwich immunofluorescent assay using europium doped nanoparticles was employed and the NGAL monoclonal antibodies (MAbs) conjugate as labels were generated by optimizing electric fusion parameters. Eighty-three urine samples were used to evaluate the clinical application efficiency of this method.

**Results:**

The quantitative detection range of NGAL in AKI was 1-3000 ng/mL, and the detection sensitization was 0.36 ng/mL. The coefficient of variation (CV) of intra-assay and inter-assay were 2.57-4.98 % and 4.11-7.83 %, respectively. Meanwhile, the correlation coefficient between europium nanoparticles-based lateral fluorescence immunoassays (EU-NPS-LFIA) and ARCHITECT analyzer was significant (R^2^ = 0.9829, *n* = 83, *p* < 0.01).

**Conclusions:**

Thus, a faster and easier operation quantitative assay of NGAL for AKI has been established, which is very important and meaningful to diagnose the early AKI, suggesting that the assay can provide an early warning of final outcome of disease.

**Supplementary Information:**

The online version contains supplementary material available at 10.1186/s12882-021-02493-w.

## Background

Coronavirus Disease 2019 (COVID-19) has widely spread in the worldwide scale with serious disaster[1].Acute kidney injury (AKI) has a higher rate of morbidity and mortality in common complication for critical illnesses and counted about 5-7 % of hospitalized patients in world[2].Several studies have evaluated the development of AKI is more strongly related to worse outcomes and mortality rates of COVID-19, described incidence of AKI that ranges widely from 0.5 to 36.6 % in COVID-19 patients[3].Early detection and precise treatments of AKI can implement better preventive strategies and prevent deterioration of renal function and renal failure, effectively contain progression of the COVID-19 hospitalized patients[4]. Among them, neutrophil gelatinase-associated lipocalin (NGAL) has been recognized as one of the promising biomarkers candidate for detection of AKI. NGAL is a 25 kDa glycoprotein associated with gelatinase from neutrophil and usually exist at lower level in human tissues such as stomach, colon and kidney, but its expression is dramatical increased in serum and urine when the kidney was with ischemic or nephrotoxic injury[5].

Lateral flow immunoassays (LFIA) has been regard as desired screening assays on account of simplicity, in-situ analysis and easy to work[6]. The LFIA with fluorescent microparticles have already been used for detection of various microbial pathogens and several inflammation markers[7, 8]. Several novel nanoparticles have been generally applied to improve the sensitivity of LFIA, including carbon nanoparticles, quantum dots, fluorescent dyes, magnetic nanolabels and europium nanoparticles (EU-NPS)[9]. EU-NPS as carriers can improve 100-fold sensitivity contrast with colloidal gold nanoparticles labeled in LFIA[10]. EU-NPS are long fluorescence lifetime and also available with an average particle size of 75–100 nm range, the large Stokes shift of which is usually over 200 nm is conducive to avoid the interference of scattered light caused by measuring excitation light. EU-NPS have wide excitation band so that it is beneficial to increase the excitation energy, the sharp emission peak, low background and high resolution, used in sandwich-type immunoassays of medical diagnostics recently[11].

Here, we established a new method with EU-NPS as labels of LFIA for the rapid, sensitive and early measurement of NGAL in urine based on two monoclonal antibodies (MAbs) 1G1 and 2F4 which are discoveried by our lab. The method is double-antibody sandwich immunofluorescent assay using EU-NPS and MAbs conjugate as labels. The mAb 1G1 was conjugated with EU-NPS and the mAb 2F4 was used to capture EU-NPS-1G1-antigen complex in T-line. Our results showed that EU-NPS-LFIA could be used for the early NGAL detection in urine and allow improvement in the treatment of AKI patients.

## Methods

### Expression and purification of NGAL

The human NGAL gene sequence from Genbank (NP_005555.2) was synthesized by Beijing Institute of Genomics (BGI) and added restriction enzymes *Hind*III and *Xho*I at both ends. The plasmids were digested with *Hind*III and *Xho*I, and then cloned into the pSecTag2A vector. The constructed plasmids were sequenced to confirm without mutation, and then transformed into Chinese Hamster Ovary (CHO) cells by the Lipofectamine^TM^2000. After 8 days, the cell supernatant was collected after filtering 0.45 μm filter. The expressed NGAL-6×His protein was purified with the Nickel Nitrilotriacetic Acid (Ni-NTA) column, and the different fractions were collected and appraised by sodium dodecyl sulfate-polyacrylamide gel electrophoresis (SDS-PAGE). The bicinchoninic acid (BCA) Protein Quantification Kit measured the concentration of protein.

The purified recombinant protein was subjected in 12 % SDS-PAGE, then adsorbed onto a polyvinylidene fluoride (PVDF) membrane. After the membrane was blocked in tris buffer with 1 % Tween-20 (TBST) solution containing 5 % skimmed milk at room temperature (RT) for 2 h, incubated with HRP-conjugated anti-6×His tag antibody (1:5000) in the dark for 1 h at RT, visualized with Benzidine after washed with TBST and visualized by Bio-Rad Western blotting detection system (DNR Bio-Imaging Systems Ltd., Israel).

### Generation and purification of MAbs

In the first immunization, the female BALB/c mice (age of 6 weeks, a total of 10) were immunized with 50 µg NGAL-6×His recombinant protein emulsified in equal dosage of complete Freund’s adjuvant, and accessional immunized were accomplished with protein emulsified in incomplete Freund’s adjuvant. Two hypodermic injections on the back of mice and subsequent were intraperitoneal injections spaced 21 days. The serum samples were collected one week after the third injection, and the titre of antiserum was determined with indirect enzyme-linked immunosorbent assay (ELISA)[12]. Three days before fusion, mice were performed to booster immunization with 50 µg of NGAL-6×His diluted with 0.9 % NaCl.

The isolated immune mice spleen cells and the SP2/0 myeloma cells mixed at a ratio of 3:1 to fuse in a platinum electrode LF498-3 fusion chamber (BEX Co., Ltd, Japan) as described literature[13]. Briefly, the mixed cell was washed twice with 10 mL electrofusion buffer (0.3 M mannitol, 0.1 mM CaCl_2_, 0.1 mM MgCl_2_, pH 7.2), re-suspended at a concentration of 2 × 10^7^ cells/mL. The fusion was completed using an alternating current voltage of 50 V at 0.8 MHz for 20 s, direct current pulse voltage of 450 V of 2 repetitions for 0.5 s, and post-fusion was 50 V at 0.8 MHz for 7 s. Finally, the electric-treated cell suspension was moved from the fusion chamber into 4.5 mL of preheated RPMI 1640 (20 % fetal bovine serum) for 30 min at 37℃, then cultured in 96-well plates and incubated with 5 % CO_2_ at 37℃. After 24 h, hypoxanthine-aminopterin-thymidine (HAT) was supplemented to each well. The Cell culture supernatants were screened by ELISA after 9 days fusion, and calculated number of hybridoma clones. The BALB/c mice which injected with paraffin oil in advance were inoculated with 1 × 10^6^ of NGAL hybridoma cells, the ascites were purified by Protein A column.

### Identification of MAbs

The immunoglobulin subclasses of antibodies were analyzed using the antibody subclass identification kit. The indirect ELISA screened the specific MAbs by using purified recombinant NGAL-6×His protein and PCT-6×His protein. The interaction between antigen and antibodies were determined with BIAcore T200 system (GE Healthcare, Stockholm, Sweden) in HBS-EP buffer (0.005 % surfactant P20, 10 mM Hepes, pH 7.4, 3 mM EDTA, 150 mM NaCl). The NGAL antigen was adsorbed on CM5 biosensor chips reaching 400–480 response units (RU) by an amine coupling kit. The antibodies (2F4 and 1G1) were diluted in HBS-EP buffer were slowly passed over the chip with 50 µL/min for 5 min, respectively, and subsequently HBS-EP buffer injected over the chip to monitor the dissociation phase for 4 min. The sensor chips were regenerated with Glycine solution (pH 3.0) following the dissociation phase. For each analyte passed over the chip, the specific responses from the antigen flow channel could subtract non-specific responses for the control flow channel. The fitted saturation binding curves were plotted based on concentrations of analyte for equilibrium binding responses to calculate KD.

Purified anti-NGAL MAbs was analyzed in 12 % SDS-PAGE under non-reducing conditions and reducing conditions. Briefly, sample was mixed with 5 x non-reducing buffer or 5 x protein loading buffer and loaded onto 12 % SDS-PAGE. The specificity of anti-NGAL MAbs was determined by Western Blot. The NGAL proteins were done to 12 % SDS-PAGE, then adsorbed onto PVDF membrane that activated by soaking in methanol for 15 s, and then subjected to the electrophoresis conditions in 100 V for 2 h. After blocking in Tris-HCl buffer with 1 % Tween-20 solution (TBST) containing 5 % skimmed milk at RT for 2 h, the membrane was incubated with mouse anti-NGAL MAbs as the primary antibodies at 4 °C. The next day, membrane was washed with TBST and incubated with anti-mouse conjugated HRP IgG in the dark for 1.5 h at RT. Finally, the membrane was visualized by the Western blot detection system of enhanced chemiluminescence (ECL).

### Competitive enzyme-linked immunosorbent assays (cELISA)

Two MAbs were tested for the ability to recognize to unique epitopes on NGAL by cELISA. MAb was conjugated with HRP by using HRP Antibody Labeling Kit (Shanghai YSRIBIO industrial co., LTD), and the working concentration of which was tested through direct ELISA. 96-well Microtitre plates were coated with 2 µg/mL of NGAL antigenin overnight at 4 °C, then unlabeled MAb (0.2 µg/ well, 2 µg/ well) was competitively bound with the optimal dilution ratio of HRP labeled MAb and incubated for 1 h at 37 °C. The enzymatic reaction was appeared with hydrogen peroxide by substrate 3,3’,5,5’-Tetramethylbenzidine (TMB) and stopped by 2 M sulfuric acid to all wells. The absorbance (OD450 nm) was determined by Bio-Rad microplate reader (Bio-Rad Laboratories, Inc). The blocking effects of MAbs was calculated by using the following equation: 100×[1-OD_450 nm_ of (HRP-MAb + MAb)/OD_450 nm_ of HRP-MAb]. Two MAbs recognized different epitopes if blocking effects observed more than 40 %.

### Conjugation of EU-NPS

The Anti-NGAL monoclonal antibody 1G1 and 2F4 were covalently conjugated to EU-NPS with standard procedure of Bangs Laboratories. Briefly, 100 µL EU-NPS were added to 900 µL 0.05 M MES (pH 7.0) and dispersed by ultrasound, vibrated for 15 min at RT in the presence of 0.08 M N-hydroxysulfosuccinimide (NHS) and 0.05 M 1-(3-Dimethylaminopropyl)-3-ethylcarbodiimide hydrochloride (EDC). The activated EU-NPS ware washed with coupling buffer (0.05 mM H_3_BO_3_, 0.04 mM Na_2_B_4_O_7_, pH 7.5) and reacted with 0.3 mg antibody for 2.5 h at RT. The europium-conjugated compound was incubated in 1000 µL blocking buffer (10 % BSA, 20 % tween-20, 0.05 M Tris-HCl) for 1 h, added to 1000 µL stock solutions (10 % BSA, 20 % trehalose, 20 % tween-20, 0.05 M Tris-HCl ) to store at 4℃.

### The development of LFIA

The LFIA strips were consisted of nitrocellulose membrane, conjugate pad, sample pad and absorbing pad. The glass fiber membranes were soaked in the blocking buffer (20 % trehalose, 10 % BSA, 20 % tween-20, 0.05 M Tris, 3.2 mM EDTA.Na_2_, pH 8.6) for 1.5 h. The concentration of 1 mg/mL MAbs (2F4 or 1G1) was coated on the test line (TL) of nitrocellulose membrane, and goat-anti-mouse IgG 1 mg/mL was coated on the same NC membrane at a distance of 4 mm to form a quality control line (CL), the spray volume of dispenser instrument was set at 1 µL/cm. The membrane and glass fiber mat were dried for 48 h at 45℃ before tested. The different EU-NPS-1G1 and EU-NPS-2F4 conjugate particles were coated on the conjugate pad, and fluorescence signal was measured by immunofluorescent analyzer (Guangzhou Labsim Biotech Co., Ltd).

### Urine sample collection and patients

The total of 83 Human urine samples from AKI patients were harvested from Affiliated Hospital of Jilin Medical University. The ethical guidelines were strictly complied in the experiment, was provided by the Affiliated Hospital of Jilin Medical University (No.2018-LW029). All subjects received oral and in written informed consent in Chinese for the study of urine samples. All experiments were performed in accordance with the Declaration of Helsinki Ethical Principles. The average age of patients was 62 years ranging from 20 to 80 years who were not infected by COVID-19 ,and AKI stage 1–3 was classified according to Kidney Disease Improving Global Outcomes AKI criteria[14]. Urine samples were collected for NGAL analysis up to 12 h before AKI was diagnosed and at frequent intervals after operation at various time points (12, 24, 48 h). For minimize potential confounding factors, urine samples were analyzed rapidly at clinical chemistry laboratory including urine biochemistry (total protein concentrations < 1 g/dL, urea < 12 g/dL, glucose < 1 g/dL, Urine Creatinine < 1 g/dL, albumin < 2.5 g/dL, pH 4.5-9.0) and carried out in duplicates. Within 30 min of samples collection, they were centrifuged at 3000 rpm at 4℃ for 15 min. A minimum of 100 µL of supernatant was dispensed into sterile containers and stored at -80℃ for further analyses, to avoid repeated freeze-thaw cycles.

The measurement of NGAL levels was performed using ARCHITECT urine NGAL reagent Kit (Lisnamuck, Longford, Co. Longford, Ireland) utilized a non-competitive, sandwich format with chemiluminescent signal detection. Urinary NGAL was recognized by anti-NGAL antibody which was covalently attached to paramagnetic particles in microparticle reagent, and the conjugate of second anti-NGAL antibody associated with acridinium. Following the manufacturer’s instructions, the calibration assay was carried out in the range of 0-1500 ng/mL and the concentration of NGAL was measured.

### Clinical sample testing and analysis

The serial concentrations of NGAL standards antigen (10, 50, 100, 200, 400, 800, 1500, 2000 and 3000 ng/mL) were prepared by using FBS to strengthen specific reaction of bioconjugate, each concentration done three replicates. After the clinical samples were added onto the sample pads, the results of fluorescence intensity on the T line (HT) and the C line (HC) were recorded by the reader. Quantitative detection was completed by the HT/HC ratio to effectively eliminate strips (T and C) difference and matrsample standard matrix effects[15]. The standard curve was plotted against each concentration of NGAL and HT/HC ratio.

### Statistical analysis

The Passing-Bablok regression analysis and Bland-Altman plot were performed by analysis of variance (ANOVA) of MedCalc and SPSS 17.0 software. All data were showed as mean value with standard deviation (mean ± S.D. ).

## Results

### Expression and purification of recombinant NGAL protein

**Fig. 1:**
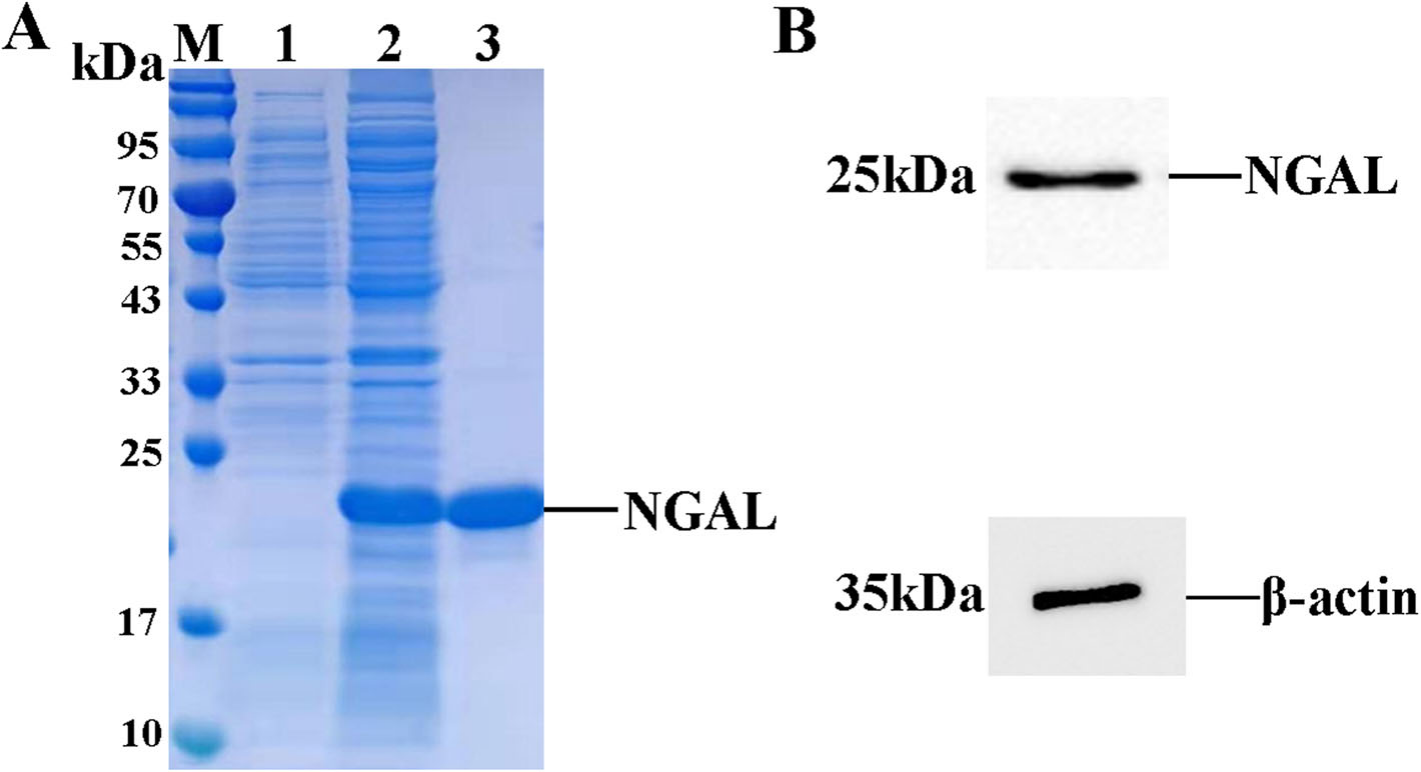
Expression and purification of NGAL-6×His.** (A)** Purification of NGAL-6×His protein were appraised by SDS-PAGE, Lane M, protein marker; Lane 1, supernatant of CHO cell culture; Lane 2, supernatant of induced sample; Lane 3, purified protein. **(B)** Western blot analysis of NGAL-6×His expression

The pSecTag2A-NGAL recombinant plasmid was transfected into CHO cells. The NGAL-6×His protein mainly expressed in cell supernatant and subsequently purified by Ni Sepharose. The purified recombinant NGAL protein was obtained about 95 % purity and analyzed by SDS-PAGE with molecular approximate weight of 23.7 kDa (Fig. 1 A). Western blot also confirmed the recombinant protein NGAL expression and purification (Fig. 1B).

### Generation of MAbs

**Fig. 2:**
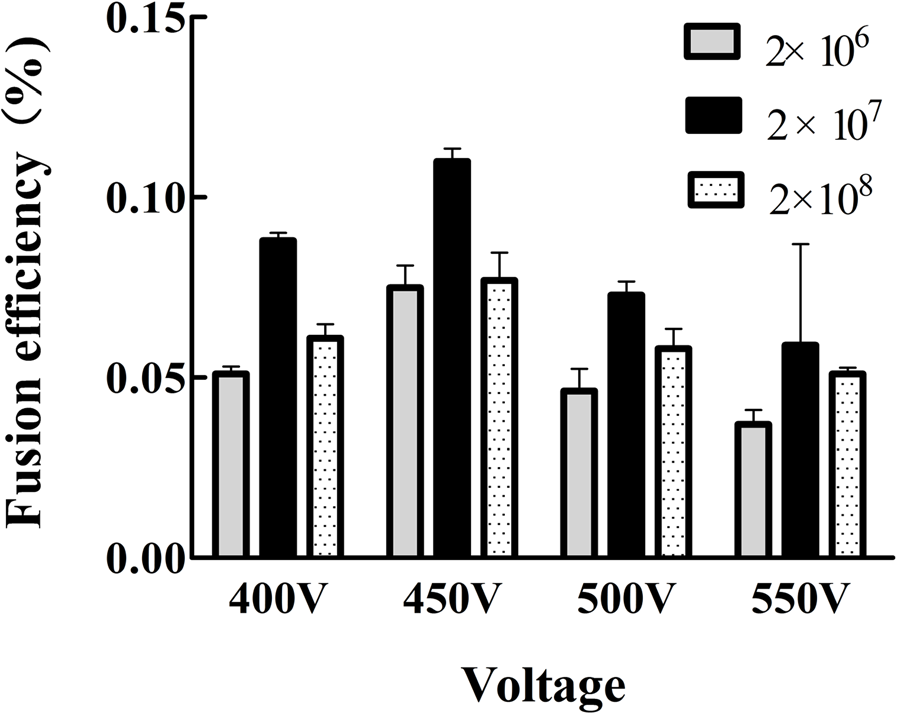
Comparison of fusion efficiency using different the puncture pulse height. The fusion cells were seeded into 96-well plates at approximately 2 × 10^5^ cells/well. A total of 480 wells was assessed for each condition. The calculated method of the fusion efficiency (%): the total number of colonies in 480 wells were counted,then divided by the number of input B cells and multiplied by 100. The columns represent the average fusion efficiency (%) of 3 experiments, the error bars represent the SD

First, we compared the effects of DC voltage on cell membrane perforation under different electric field intensities, and the pulse amplitude was 400 V, 450 V, 500 and 550 V respectively, so as to optimize the electric fusion scheme. According to previous reports, cell concentration has a significant impact on fusion efficiency[16]. The mixed suspension of isolated spleen cells and SP2/0 myeloma cells was transferred into the fusion chamber at different concentrations of approximately 2 × 10^6^, 2 × 10^7^, and 2 × 10^8^ cells /mL. The fusion efficiency was the highest at 450 V DC, and the optimal cell concentration was 2 × 10^7^ cells/mL (Fig. 2).

### Characterization of MAbs

**Fig. 3:**
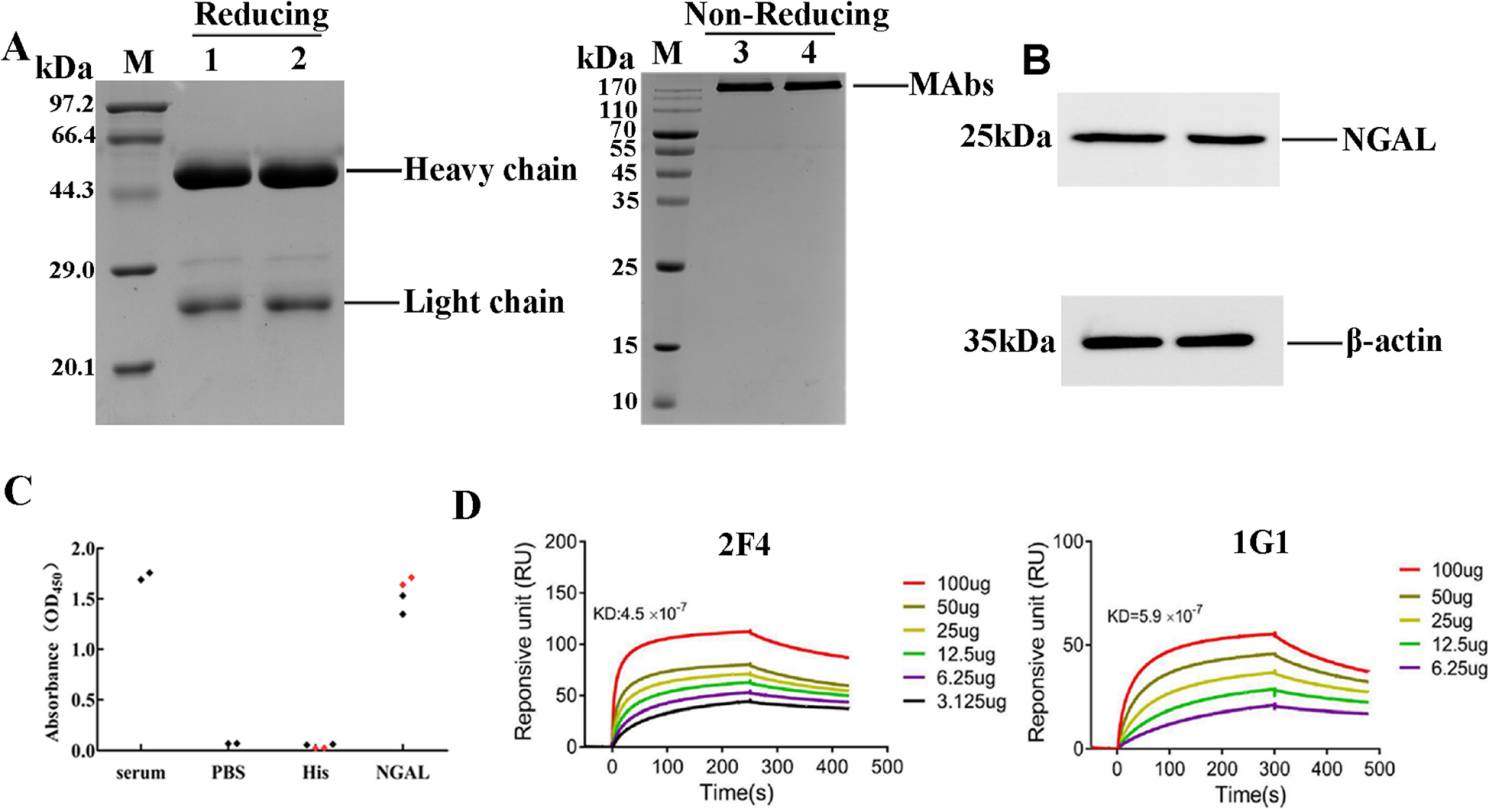
Characterization of MAbs. **(A)** The ascites were assessed by SDS-PAGE under non-reducing and reducing, Lane M, protein marker; Lane 1 and 3, purified the ascites of 2F4; Lane 2 and 4, purified the ascites of 1G1.**(B)** The ascites were assessed by Western blot, Lane 1, Western blot analysis of mAb 2F4; Lane 2, Western blot analysis of mAb 1G1. **(C)** The Cross-reactivity of 2 hybridoma lines was tested by Indirect ELISA, NGAL-6×His and PCT-6×His were coated onto microtiter plates. The positive control was a NGAL-6×His immune serum; PBS were used as blank control. Red and black dots were meant 1G1 and 2F4 ELISA OD450 nm values, respectively. **(D)** Relative affinity of MAb 2F4 and 1G1

The MAbs were purified by Protein A column. The purified MAbs were separated by 12 % SDS-PAGE, two bands with molecular weight 55 kDa and 25 kDa were observed under reducing condition. The clear bands of 170 kDa were noted under non-reducing condition, which are the intact protein of 2F4 and 1G1**(**Fig. 3 A). Western blot indicated that all MAbs specifically bind to human NGAL protein **(**Fig. 3B). After cell electrofusion and sub-cloning, the supernatants of hybridomas were detected by indirect ELISA with NGAL-6×His and PCT-6×His to ensure antibodies specific binding to the NGAL. Two highly positive hybridomas (1G1, 2F4) with specific NGAL binding while without cross-reaction to PCT-6×His were successfully selected for production and purification of MAbs (Fig. 3 C). To detect the affinity of two MAbs, the interaction between antibodies with different concentrations and NGAL protein was analyzed by BIAcore T200 system. The kinetic diagram showed that the affinity of 2F4 and 1G1 were 4.5 × 10^− 7^ and 6.0 × 10^− 7^, respectively (Fig. 3D). The isotypes of those two MAbs which were detected by commercial kits were IgG1.The blocking rate of 2F4 and 1G1-HRP pairing was 63 % by cELISA detection, indicating that they recognized different epitopes.

### EU-NPS-LFIA Procedures

**Fig. 4:**
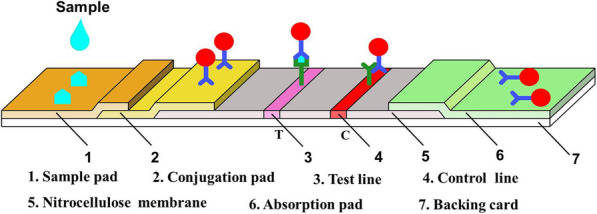
Schematic description of fluorescence immunoassay system employed Europium-conjugated NGAL MAbs

Based on EU-NPS as labels and the sandwich-type immunoassay, the LFIA was established for detection of NGAL. MAb 2F4 was coated on nitrocellulose membrane and paired with EU-NPS-1G1 based on antibodies pairing in lateral flow immunochromatography platform. As schematically illustrated (Fig. 4), TL and CL of nitrocellulose membrane were coated with the MAbs 2F4 and goat anti-mouse IgG. EU-NPS-1G1 was labeled on the conjugate pad. The sample containing NGAL antigen migrated towards the conjugate pad to combine with EU-NPS-1G1 and form antigen-antibody complexes. Subsequently, the complexes were captured by mAb 2F4 in T-line while migrating to form sandwich complexes. Excess complex was combined the goat anti-mouse IgG. Test strips were measured with a fluorescence detector after 15 min.

### Performance evaluation

**Fig. 5:**
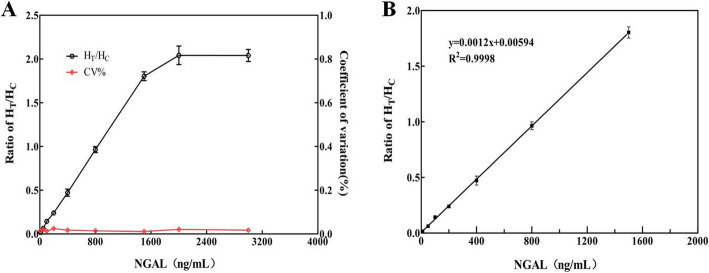
Standard curves for NGAL. (**A**) The ratio of HT/HC, by antibody pairing in LFIA, the antibody pairs 2F4-labled1G1 ratio of HT/HC rose with increasing concentration of NGAL. (**B**) The standard curve of NGAL

The antibody pairs (2F4- labled1G1) as detector and capture antibodies were used to establish fluorescent immunochromatographic, and the analytical performances of EU-NPS-LFIA was evaluated by building the standard curve. The relative fluorescence intensity ratio(HT/HC) was increased with NGAL concentration. EU-NPS-1G1 showed reaction to mAb 2F4 (Fig. 5 A). The high-dose hook influence wasn’t detected when concentration of antigen reached 3000 ng/mL. The regression equation was exhibited as follows: y = 0.0012 x + 0.0059 (R^2^ = 0.99), where y represents the ratio of HT/HC, x represents the concentrations of NGAL (Fig. 5B).The detection limit (LOD) was 0.36 ng/mL (3 times the standard deviation of the blank, *n* = 20) calculated by the Clinical Laboratory Standards Institute (CLSI) Guideline EP17-A2, the NGAL concentration had linear relationship in the range of 1-1500 ng/mL[17].

### Precision

Recovery experiments of the intra-assay and inter-assay were performed to evaluate precision and reproducibility of immunological method. The different concentrations (50, 200 and 800 ng/mL) of NGAL standard substance were added to negative urine samples. The intra-assay precision was calculated with three replicates at each spiked concentration in 1 day, and inter-assay precision was calculated with three replicates at each spiked concentration at every three days for fifteen days continuously[18]. The results were shown in Table 1, the calculated intra-assay coefficient of variation (CV) ranged from 2.57 to 4.98 % (n = 10), lower than 10 %. The inter-assay CV ranged from 4.11 to 7.83 % (n = 15), lower than 10 % too.

Intra-assay and inter-assay precision were verified by two evaluator devices (Guangzhou Wondfo Biotech Co.,Ltd and Guangzhou Labsim Biotech Co., Ltd), and the result is eventually consistent (Supplement data). These results explained that the precision of the developed LFIA was a high level, and reproducibility was an acceptable level.

**Table.1:** Reproducibility analysis of the EU-NPS-LFIA test strip by intra-assay and inter-assay precision

NGAL (ng/mL)	Intra-Assay Precision (n = 10)		Inter-Assay Precision (n = 15)	
	Mean ± SD (ng/mL)	CV (%)	Mean ± SD (ng/mL)	CV (%)
50	49.57 ± 0.003	4.98	51.28 ± 0.037	5.68
200	199.18 ± 0.009	3.84	202.78 ± 0.019	7.83
800	799.76 ± 0.025	2.57	801.68 ± 0.039	4.11

### Specificity

The specificity of the test strips was evaluated by adding endogenous substances in normal and different concentrations urine samples, including creatinine, glucose and urea nitrogen. As shown in Table 2, the results indicated that all relative deviations (RD) was in the range of ± 10 %, suggesting antigen-antibody interaction were stable and illustrating the specificity of test strips was acceptable toward NGAL.

**Table.2:** The specificity study of the EU-NPS-LFIA with different interfering endogenous substances

Interfering Substance	NGAL (27.64ng/mL)		NGAL (53.72ng/mL)	
	Value	RD (%)	Value	RD (%)
control	0.037 ± 0.002	0.54	0.069 ± 0.003	2.35
Creatinine(10 mg/mL)	0.035 ± 0.008	-4.55	0.067 ± 0.009	-2.06
glucose(10 mg/mL)	0.036 ± 0.007	-2.55	0.071 ± 0.006	3.83
urea nitrogen(100 mg/mL)	0.036 ± 0.004	2.72	0.064 ± 0.008	-5.98

Note: RD = (Value-Standard value) / Standard value

### Clinical samples tests

**Fig. 6:**
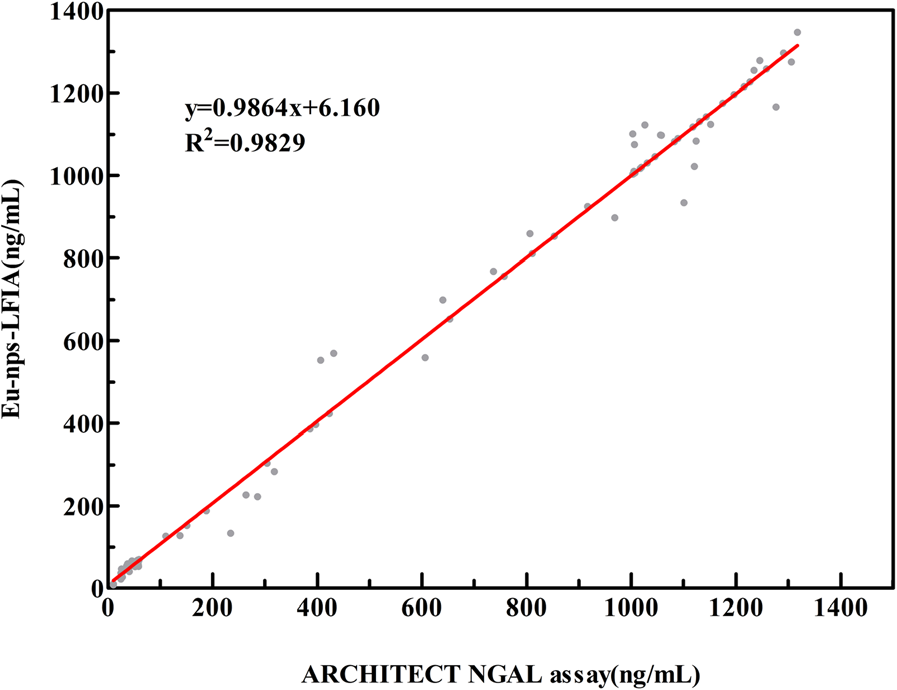
Comparison of EU-NPS-LFIA with ARCHITECT urine assay estimated correlation of the results for NGAL clinical test

In order to appraise applicative competence of the NGAL based on EU-NPS-LFIA for determination of clinical samples, a total of 83 urine samples containing 26 low value samples (11–70 ng/mL), 19 median value samples (110–800 ng/mL), 38 high value samples (800–1740 ng/mL), were measured on the ARCHITECT urine NGAL assay. The results of correlation coefficient (R^2^) of the regression curve was 0.9829 (*p* < 0.01), indicating that the two detection methods had a significant linear relationship (where x represents concentrations of NGAL obtained by the ARCHITECT analyzer, y represents values measured by developed test strips) (Fig. 6). Thus, the developed EU-NPS-LFIA for determination of NGAL was very accurate in clinical testing.

## Discussion

The EU-NPS-LFIA for detection of NGAL in human urine and diagnosis of AKI has been developed in our study. Current diagnosis of AKI is confirmed by the concentration of serum creatinine (sCr), which is steady unless at least 50 % of damaged kidney function[19]. AKI developed in 89 % of severe patients with COVID-19, and a majority of patients had predominantly oliguria when appears earlier than plasma Creatinine[20]. Biomarkers increased elevated levels at admission, which associated with increased mortality. NGAL is one of these biomarkers, levels of which may positive correlation with risk for mortality in COVID-19 hospitalized patients[21]. In AKI, several studies have discovered NGAL concentration in urine is significantly associated with sCr concentration[22]. In recent years, various electrochemical and immunological methods have been developed for the detection of NGAL, instance of electrochemical determination, solid-phase proximity ligation assay and enzyme-free electrochemical immunoassay[23, 24]. However, these techniques require precise equipment, specialized personnel and professional interpretation of the results. Therefore, to develop an effective and convenient detection assay for NGAL concentration in the urine is critical for this disorder monitoring. The low efficiency of polyethylene glycol (PEG) fusion in conventional methods to causes difficulties in obtaining functional antibodies, we optimized electric fusion parameters that enabled enhancement of fusion efficiency to prepare of viable hybridomas, and obtained two anti-NGAL MAbs. MAbs are not only high affinity, but also directed towards different epitopes, used to establish high quality of diagnostic assays.

In recent years, many studies have been reported to develop several NGAL rapid diagnostic immunoassays, including the photoelectrochemical immunosensor and three electrochemical immunosensors, the solid-phase proximity ligation assay and lateral flow assay[25]. In one study, immunosensor has been made by NGAL capture antibodies immobilized to screen-printed-modified carbon electrode and labeled addition of secondary antibody against NGA to PB-NP-decorated g-C_3_N_4_ nanosheets forming a sandwich on the SPCE[24]. The other study developed antibody against NGAL was immobilized on a screen printed electrode (SPCE) modified with electropolymerized aniline deposited on top of an electrosprayed graphene/poly-aniline (G/PANI)[25]. Two reports showed the LOD varied widely from 0.6 pg/mL to 21.1 ng/mL, depending on the g-C_3_N_4_ nanosheets with N element enhanced electrocatalytic efficiency of the nanohybrids than graphene nanosheets, and the LOD of the NGAL assay varies depending on immunosensor, conjugated-complex and antibodies[26]. According to those studies, we enlightened that sensitivity of LFIA could be improved by basing on different fluorescence nanoparticles mentioned in the preamble section.The high sensitivity nanoparticles labeled in LFIA as fluorescent probes has been continually researched over past decades, gold nanoparticles are the most widely used, but the application of the lateral flow tests based on traditional labels is limitedfor there poor identification and weaker the signal[27]. Currently there are antibodies available for the development of NGAL have been applied to different platforms for NGAL detection, but not available in LFIA based on EU-NPS labels experimented on the detection of NGAL in addition to UCP technology-based lateral flow assay [28, 29].

Fluorescence immunoassays have advantange in various detection fields, especially in mature quantum dots, that sensitivity was influenced by the MAbs and fluorescent materials, and could greatly improve by EU-NPS and specific MAbs to use the fluorescent probes[30]. The EU-NPS were applied to fluorescence LFIA strips had matured in the detection field, and enhanced several advantages of LFIA for assay sensitivity, specificity and stability[31]. Due to excellent properties of the EU-NPS that the limit of detection for NGAL in this method was 0.36 ng/mL, while the mean urine NGAL levels was about 7.0 ng/mL in healthy individuals[32]. The developments and application about the EU-NPS-LFIA of NGAL are still in initial phase, this assay need to test NGAL of serum and urine samples with COVID- 19 patients for which improvement in the treatment in the future of works.

## Conclusions

In the study, we successfully got two mouse anti-NGAL MAbs (2F4, 1G1) and were labeled with EU-NPS to establish the lateral flow immune technique. The EU-NPS-LFIA was found to detect NGAL in a wide range of 1-3000 ng/mL within 15 min, the detection sensitivity reached 0.36 ng/mL. These anti-NGAL MAbs could be reliability utilized in fluorescence LFIA of NGAL detection in the urine samples, so that it should be applicable in the AKI diagnosis.

## Supplementary information

Additional file 1 zip

## Data Availability

The datasets used and/or analyzed during the current study are available from the corresponding author on reasonable request.

## Abbreviations

COVID-19: Coronavirus Infection Disease 2019
AKI: Acute kidney injury
SCr: serum creatinine
NGAL: neutrophil gelatinase-associated lipocalin
LFIA: Lateral flow immunoassays
EU-NPS: europium nanoparticles
ELISA: enzyme-linked immunosorbent assay
MAbs: monoclonal antibodies
