# Supplementary figures and images for: Development of a europium nanoparticles lateral flow immunoassay for NGAL detection in urine and diagnosis of acute kidney injury
Source: BMC Nephrol. 2022 Jan 14;23:30. doi: 10.1186/s12882-021-02493-w (PMC8758895; doi:10.1186/s12882-021-02493-w)

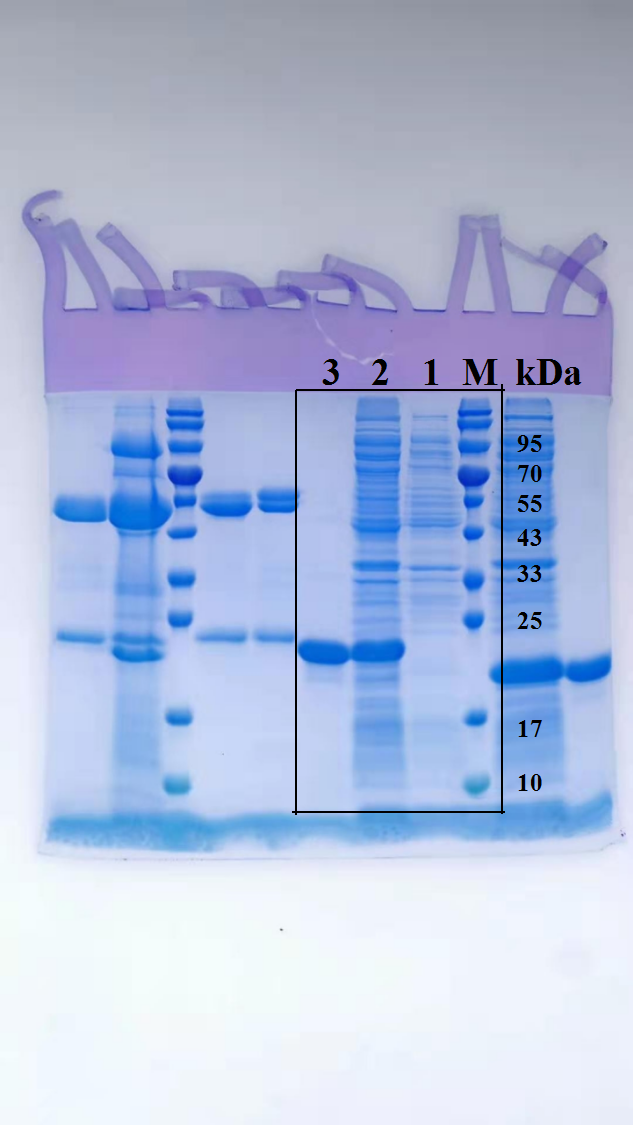

Supplement: Supplementary file 1 — zip [file 12882_2021_2493_MOESM1_ESM.zip › Figure 1A.png]

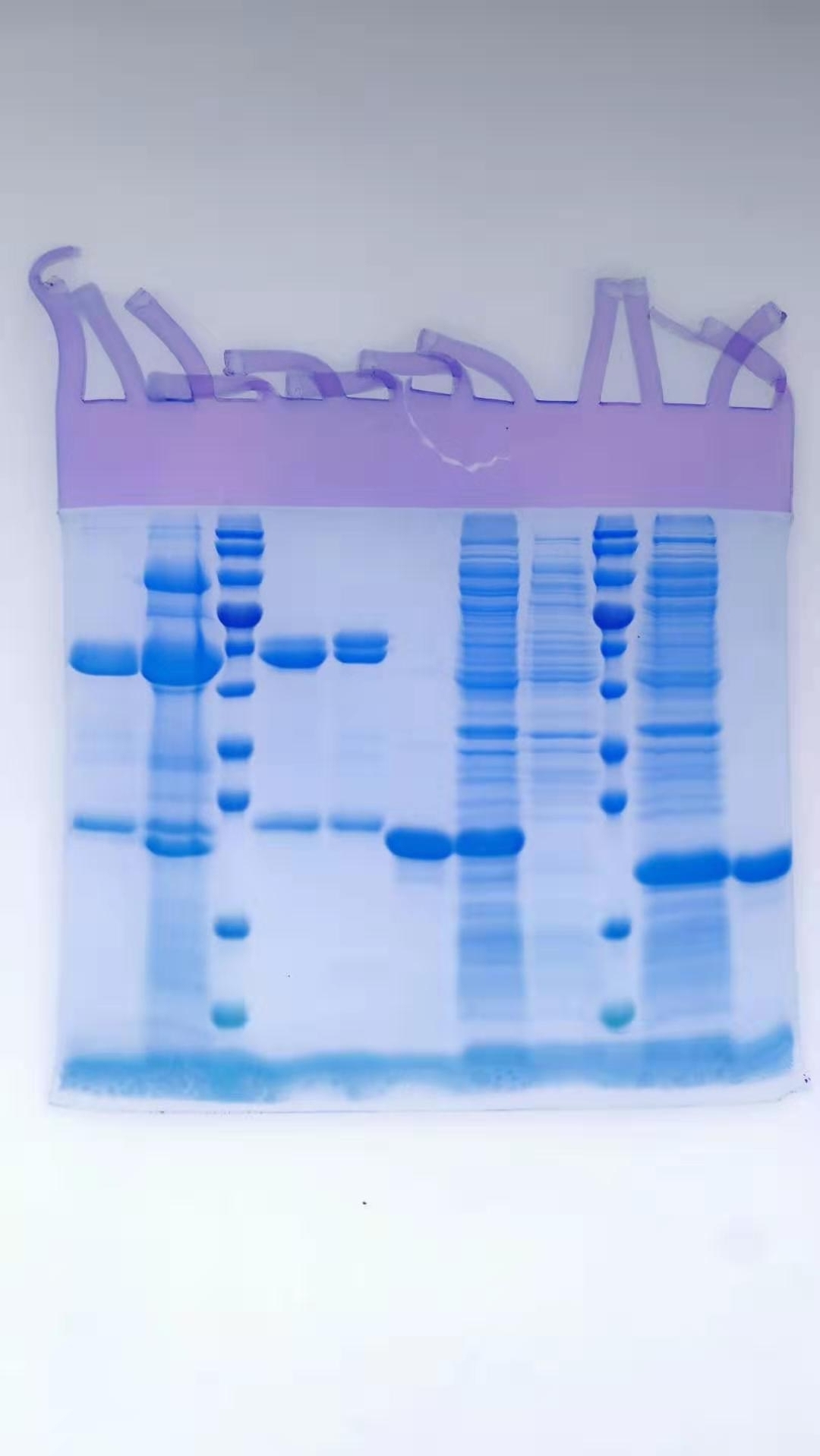

Supplement: Supplementary file 1 — zip [file 12882_2021_2493_MOESM1_ESM.zip › Figure 1A-original.JPG]

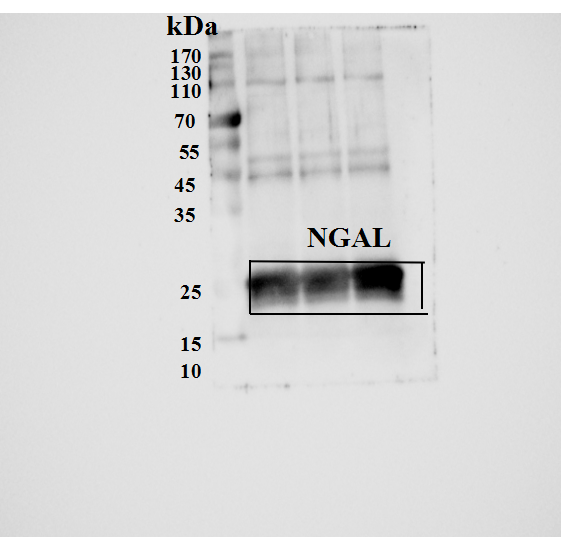

Supplement: Supplementary file 1 — zip [file 12882_2021_2493_MOESM1_ESM.zip › Figure 1B.png]

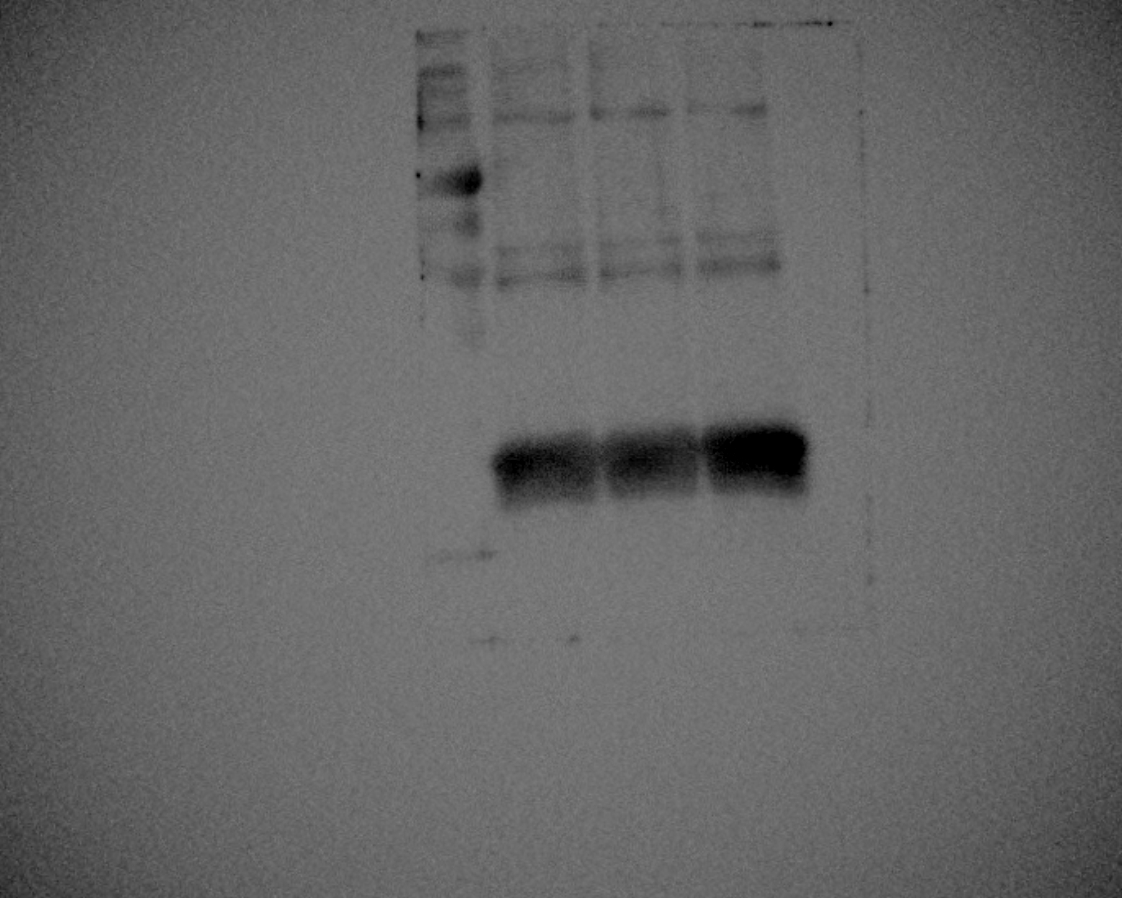

Supplement: Supplementary file 1 — zip [file 12882_2021_2493_MOESM1_ESM.zip › Figure 1B-original-1.jpg]

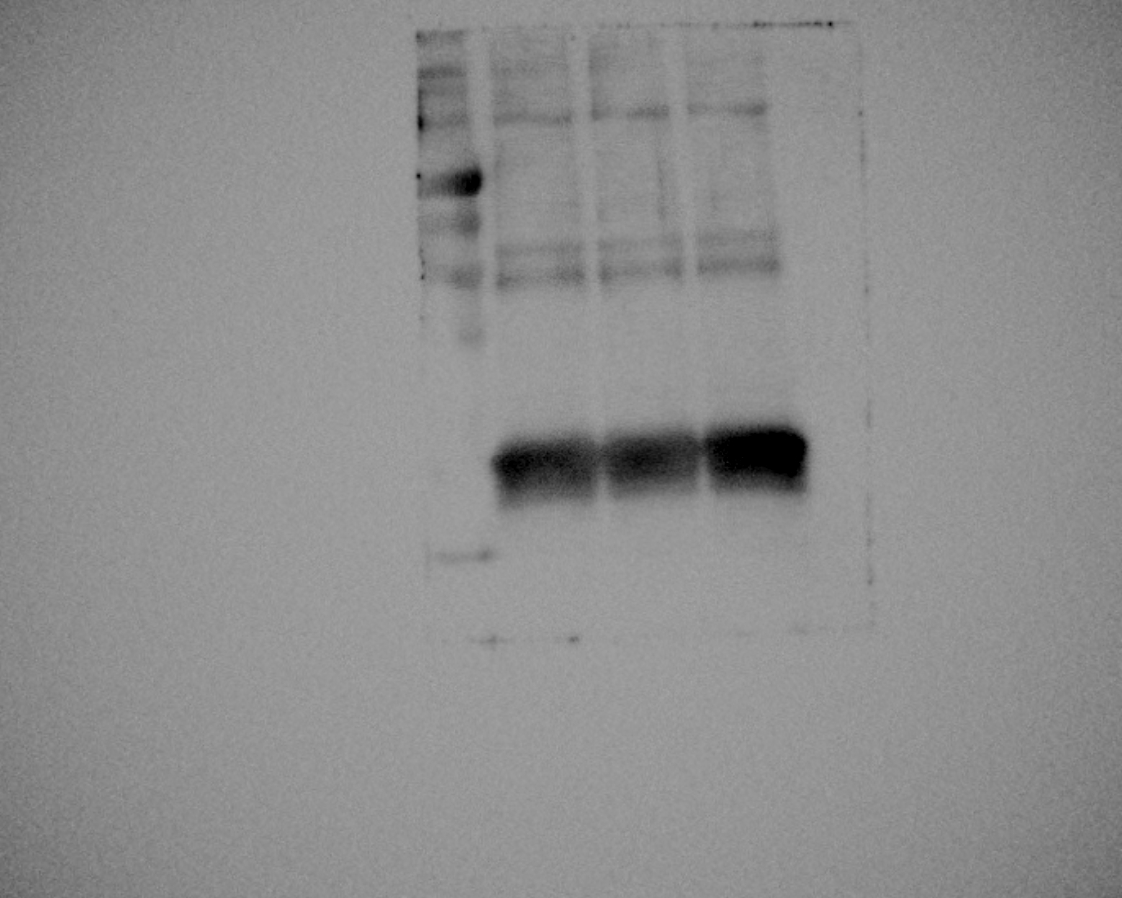

Supplement: Supplementary file 1 — zip [file 12882_2021_2493_MOESM1_ESM.zip › Figure 1B-original-2.jpg]

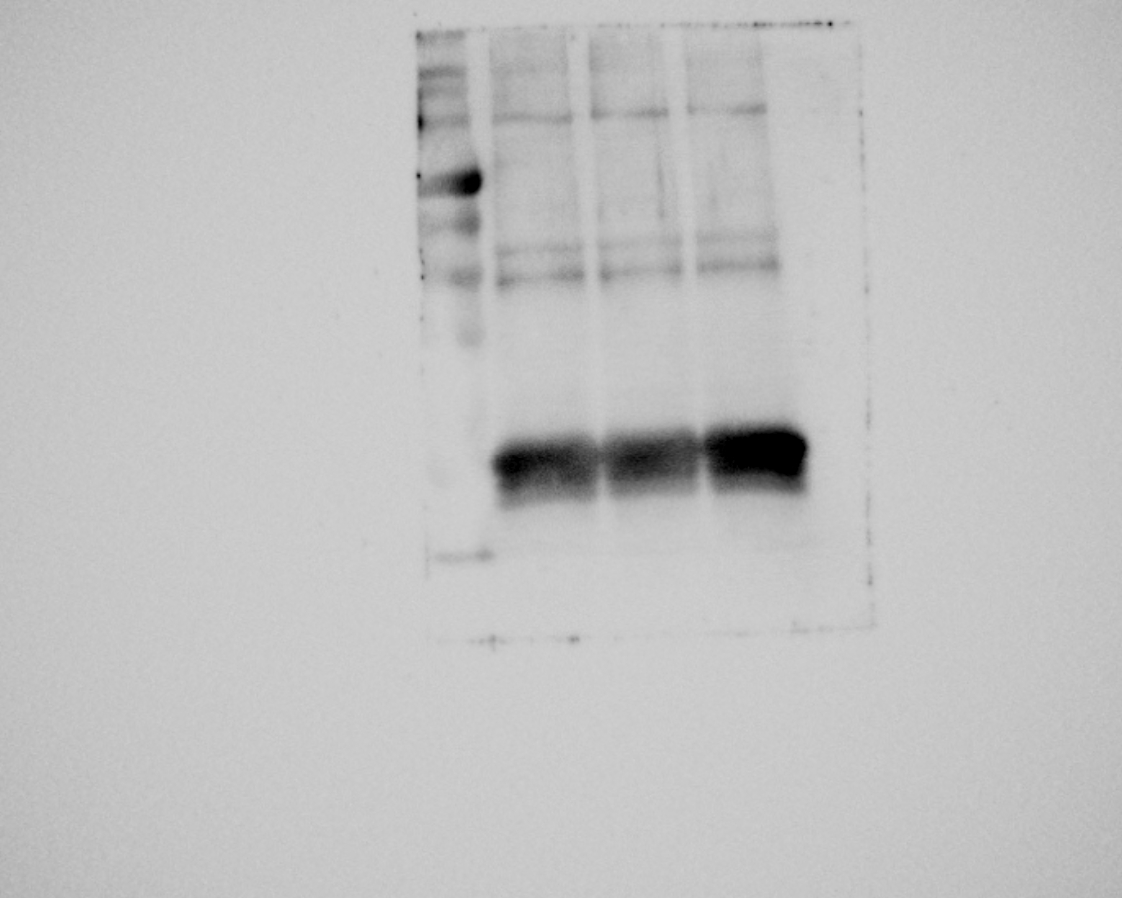

Supplement: Supplementary file 1 — zip [file 12882_2021_2493_MOESM1_ESM.zip › Figure 1B-original-3.jpg]

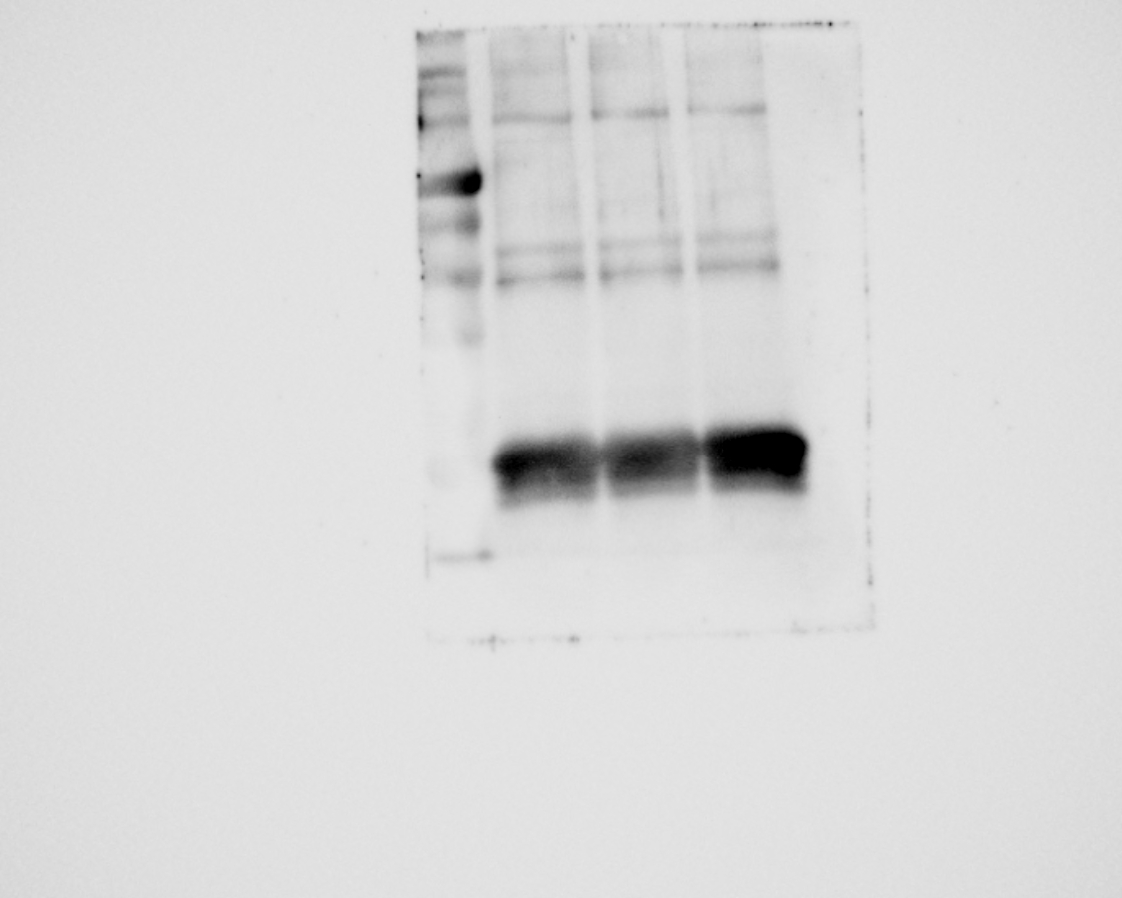

Supplement: Supplementary file 1 — zip [file 12882_2021_2493_MOESM1_ESM.zip › Figure 1B-original-4.jpg]

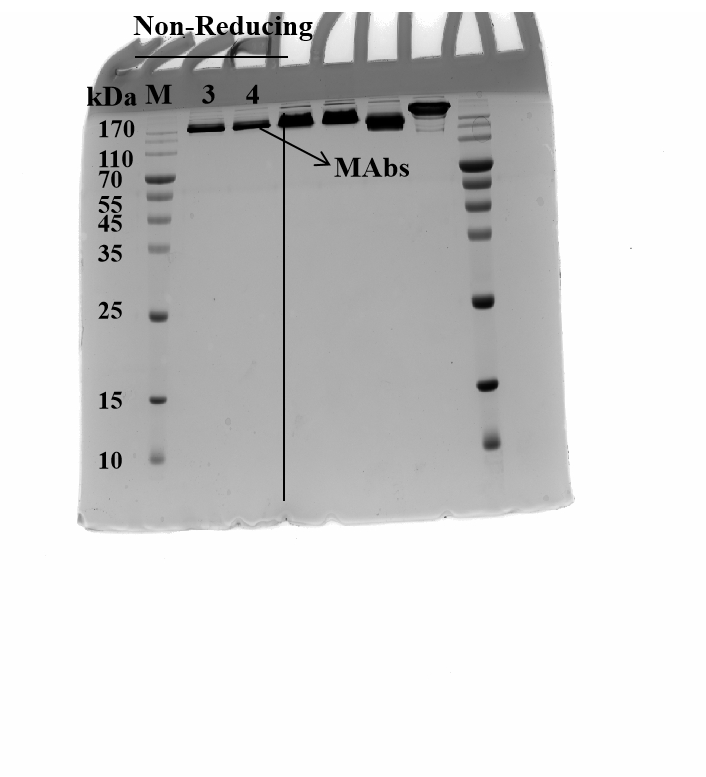

Supplement: Supplementary file 1 — zip [file 12882_2021_2493_MOESM1_ESM.zip › Figure 3A Non-Reducing.png]

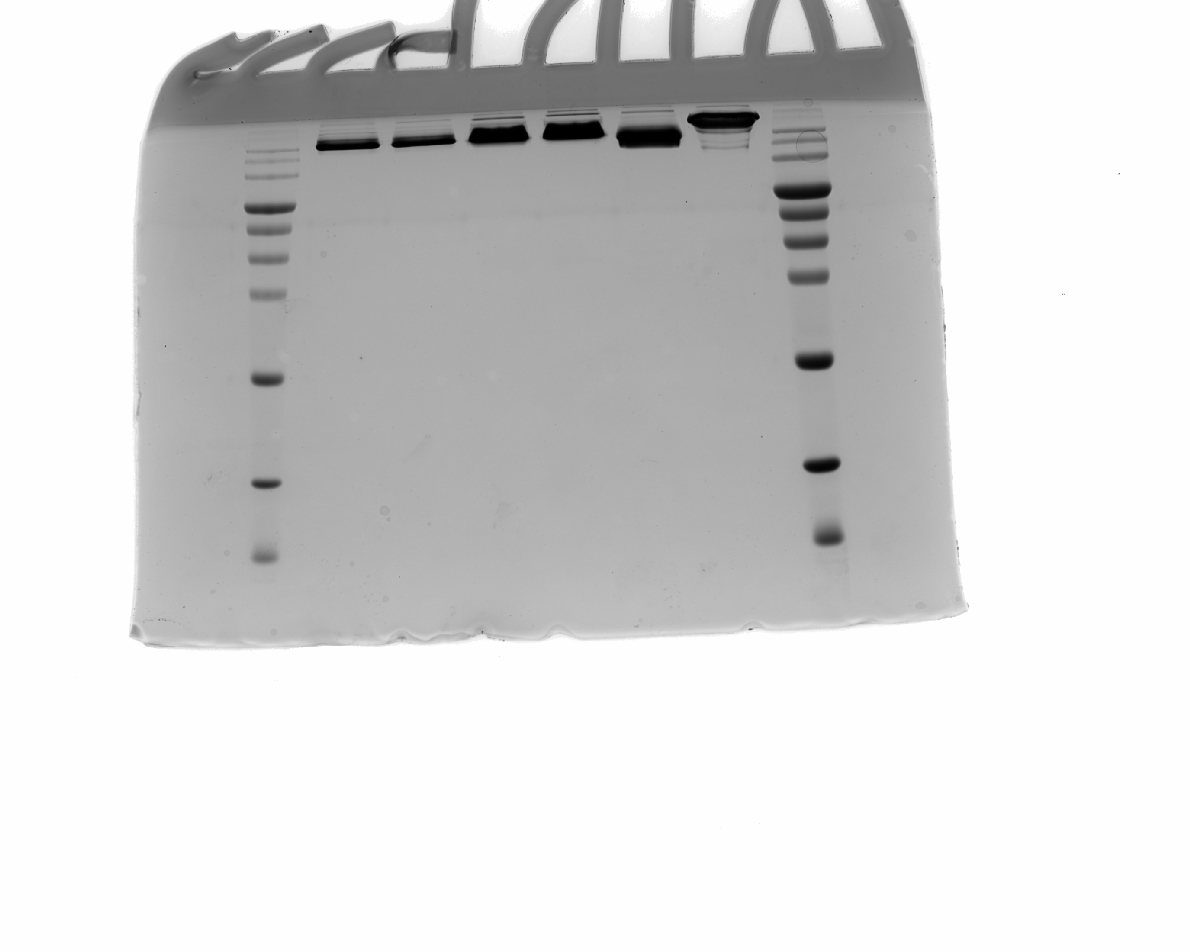

Supplement: Supplementary file 1 — zip [file 12882_2021_2493_MOESM1_ESM.zip › Figure 3A Non-Reducing-original.jpg]

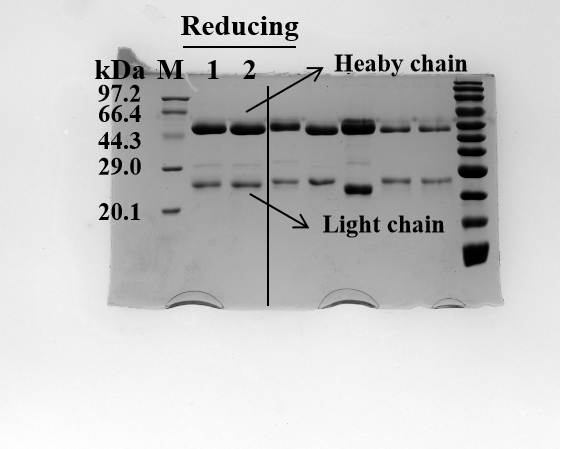

Supplement: Supplementary file 1 — zip [file 12882_2021_2493_MOESM1_ESM.zip › Figure 3A Reducing.png]

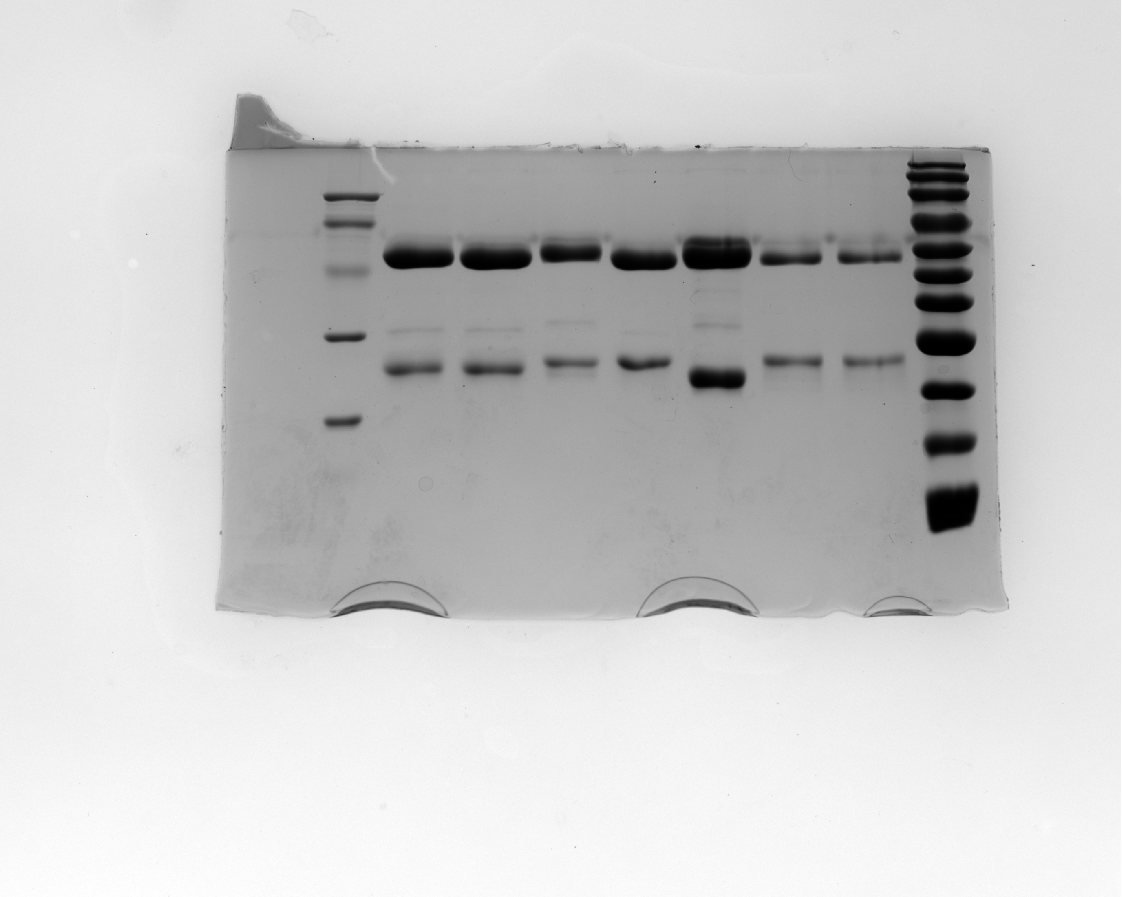

Supplement: Supplementary file 1 — zip [file 12882_2021_2493_MOESM1_ESM.zip › Figure 3A Reducing-original.tif]

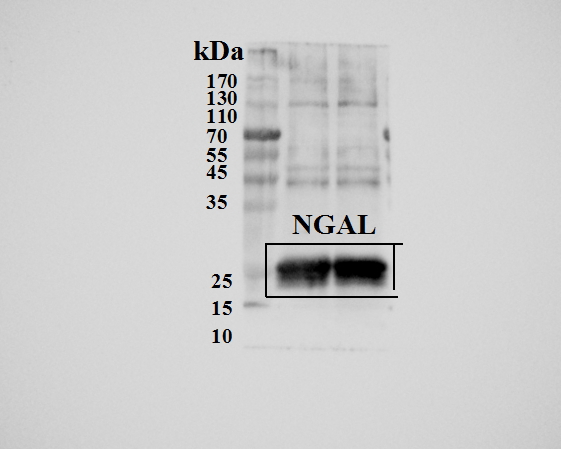

Supplement: Supplementary file 1 — zip [file 12882_2021_2493_MOESM1_ESM.zip › Figure 3B-Lane1-2F4.png]

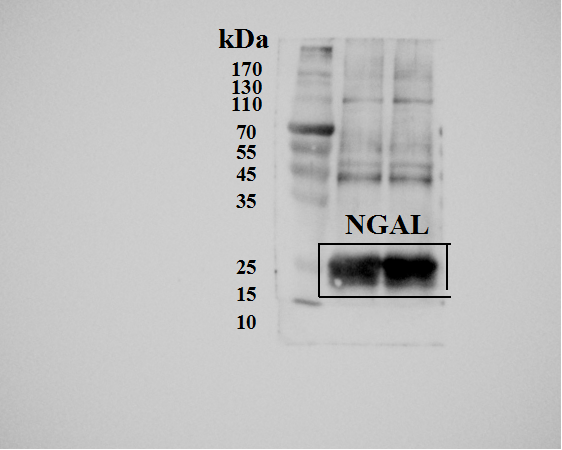

Supplement: Supplementary file 1 — zip [file 12882_2021_2493_MOESM1_ESM.zip › Figure 3B-Lane2-1G1.png]

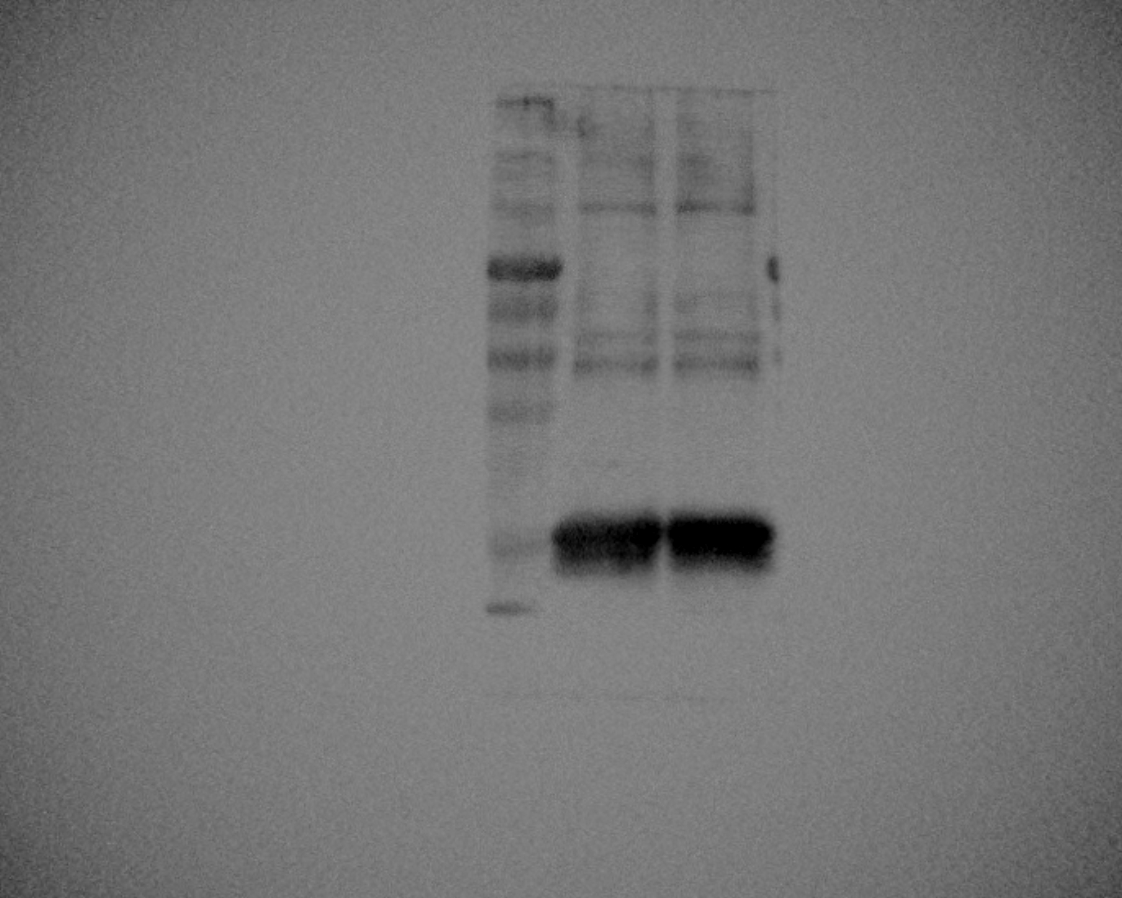

Supplement: Supplementary file 1 — zip [file 12882_2021_2493_MOESM1_ESM.zip › Figure 3B-original-Lane1-2F4-1.jpg]

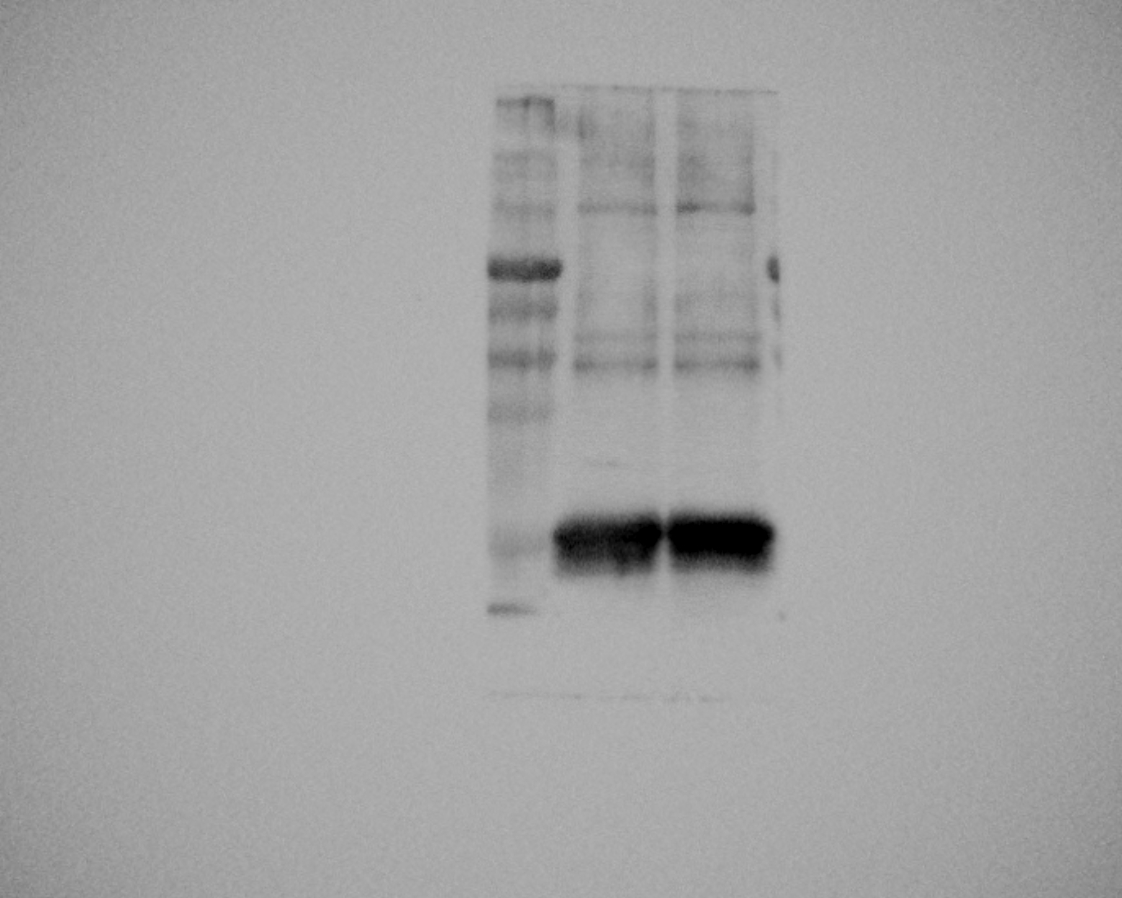

Supplement: Supplementary file 1 — zip [file 12882_2021_2493_MOESM1_ESM.zip › Figure 3B-original-Lane1-2F4-2.jpg]

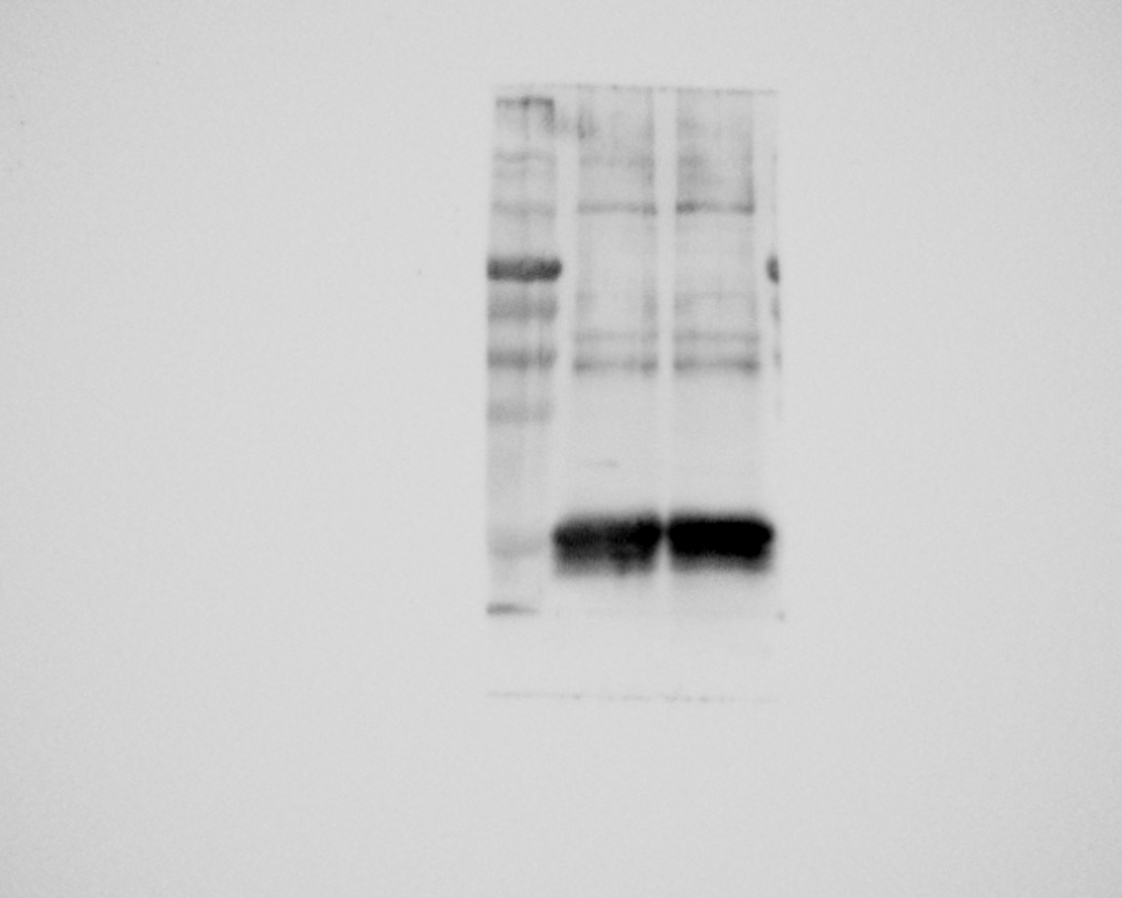

Supplement: Supplementary file 1 — zip [file 12882_2021_2493_MOESM1_ESM.zip › Figure 3B-original-Lane1-2F4-3.jpg]

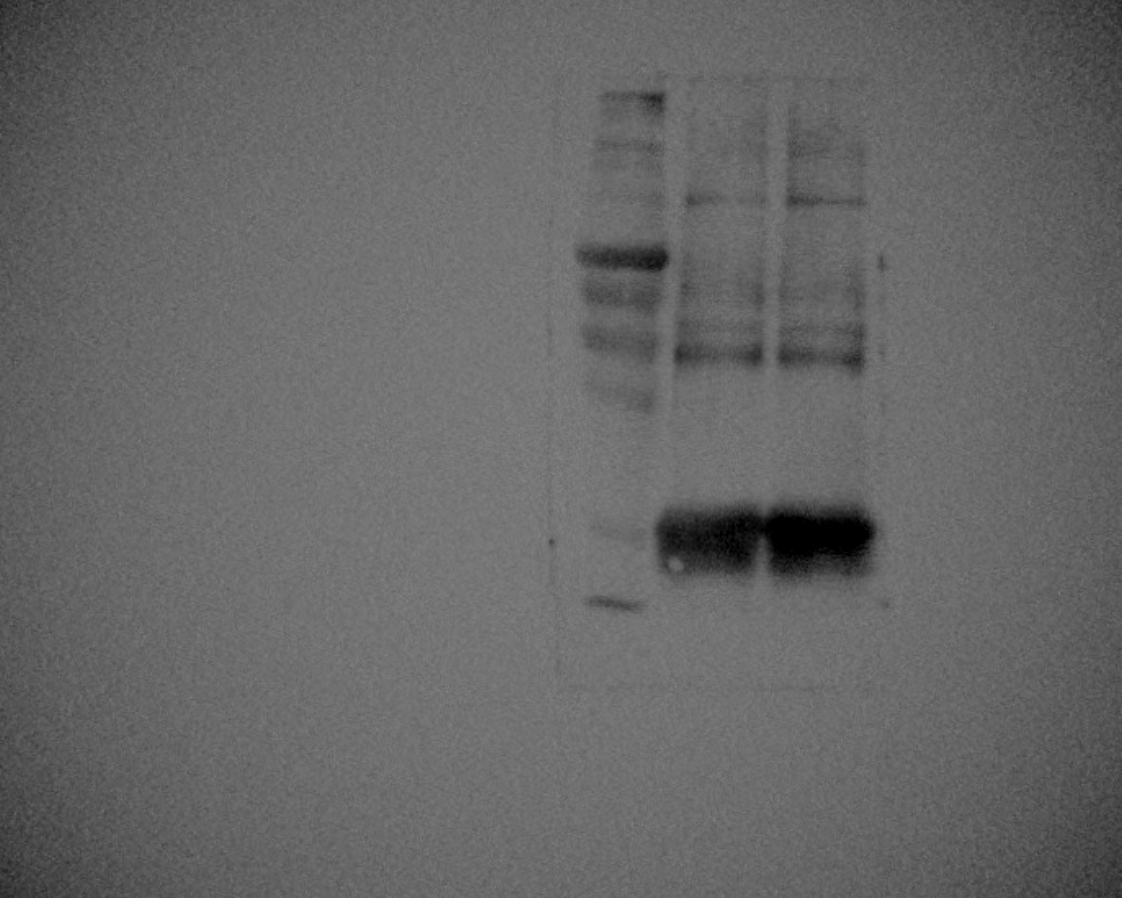

Supplement: Supplementary file 1 — zip [file 12882_2021_2493_MOESM1_ESM.zip › Figure 3B-original-Lane2-1G1-1.tif]

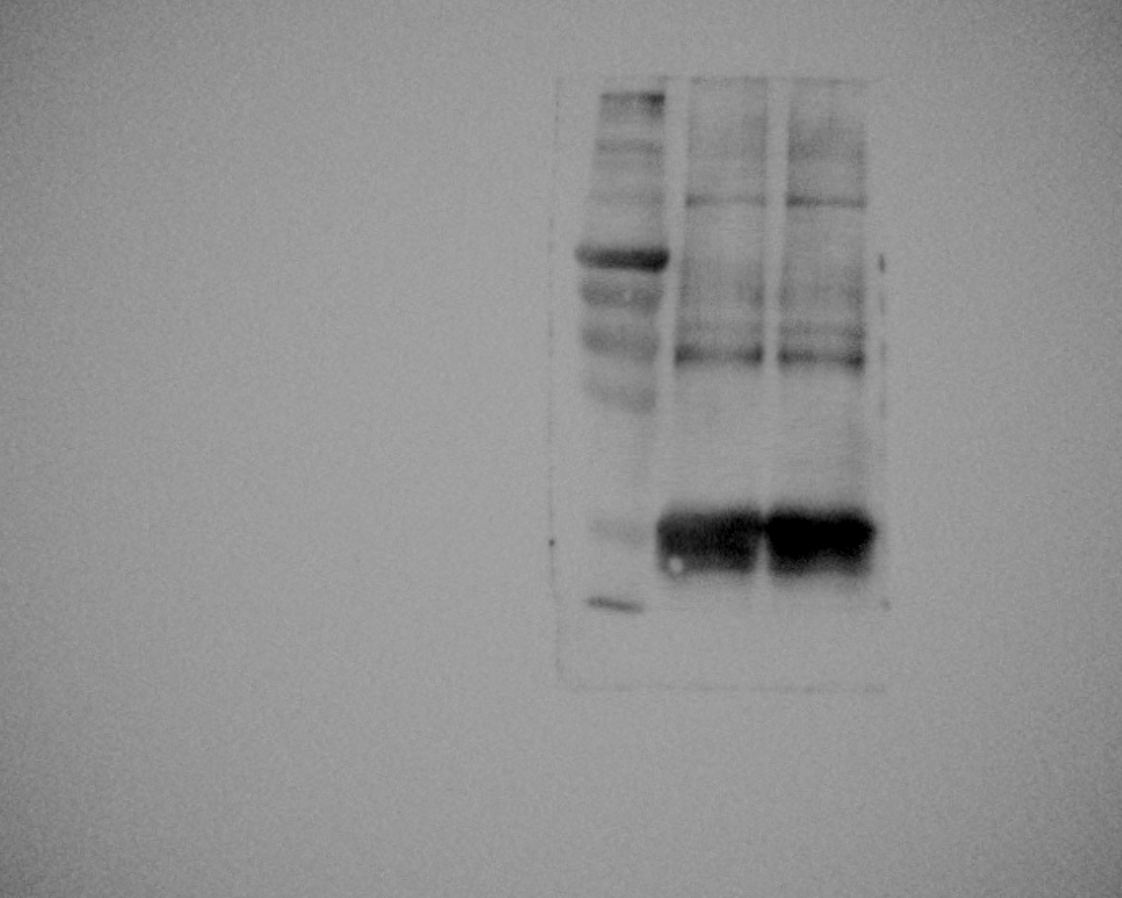

Supplement: Supplementary file 1 — zip [file 12882_2021_2493_MOESM1_ESM.zip › Figure 3B-original-Lane2-1G1-2.tif]

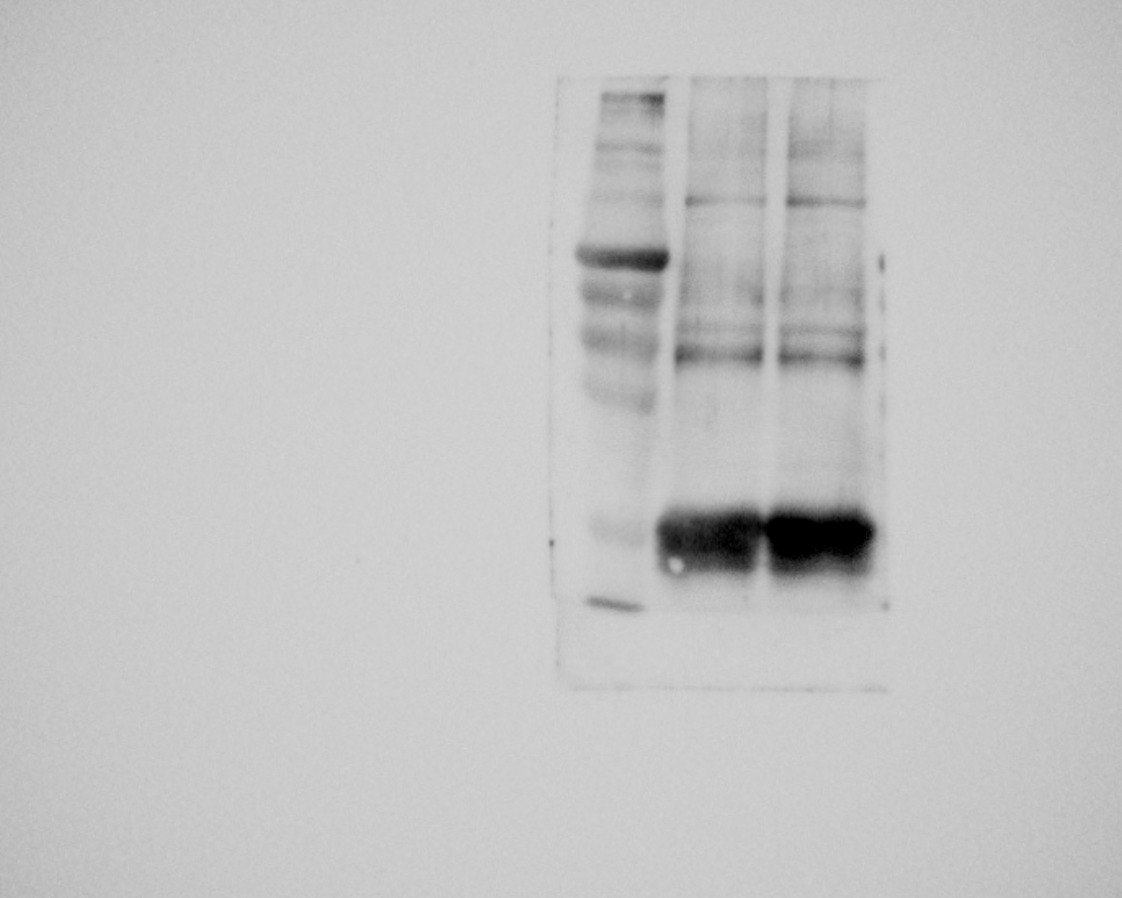

Supplement: Supplementary file 1 — zip [file 12882_2021_2493_MOESM1_ESM.zip › Figure 3B-original-Lane2-1G1-3.tif]

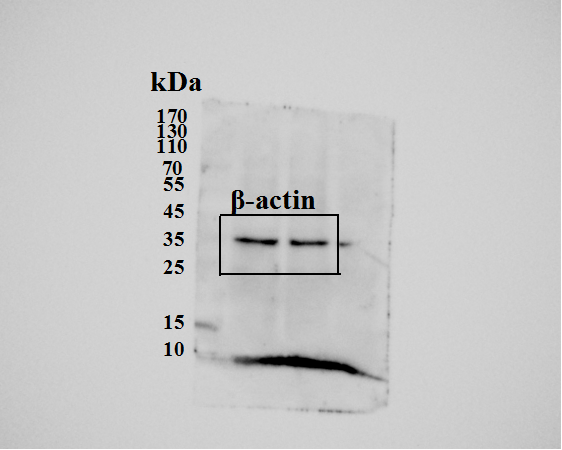

Supplement: Supplementary file 1 — zip [file 12882_2021_2493_MOESM1_ESM.zip › a┬-actin.png]

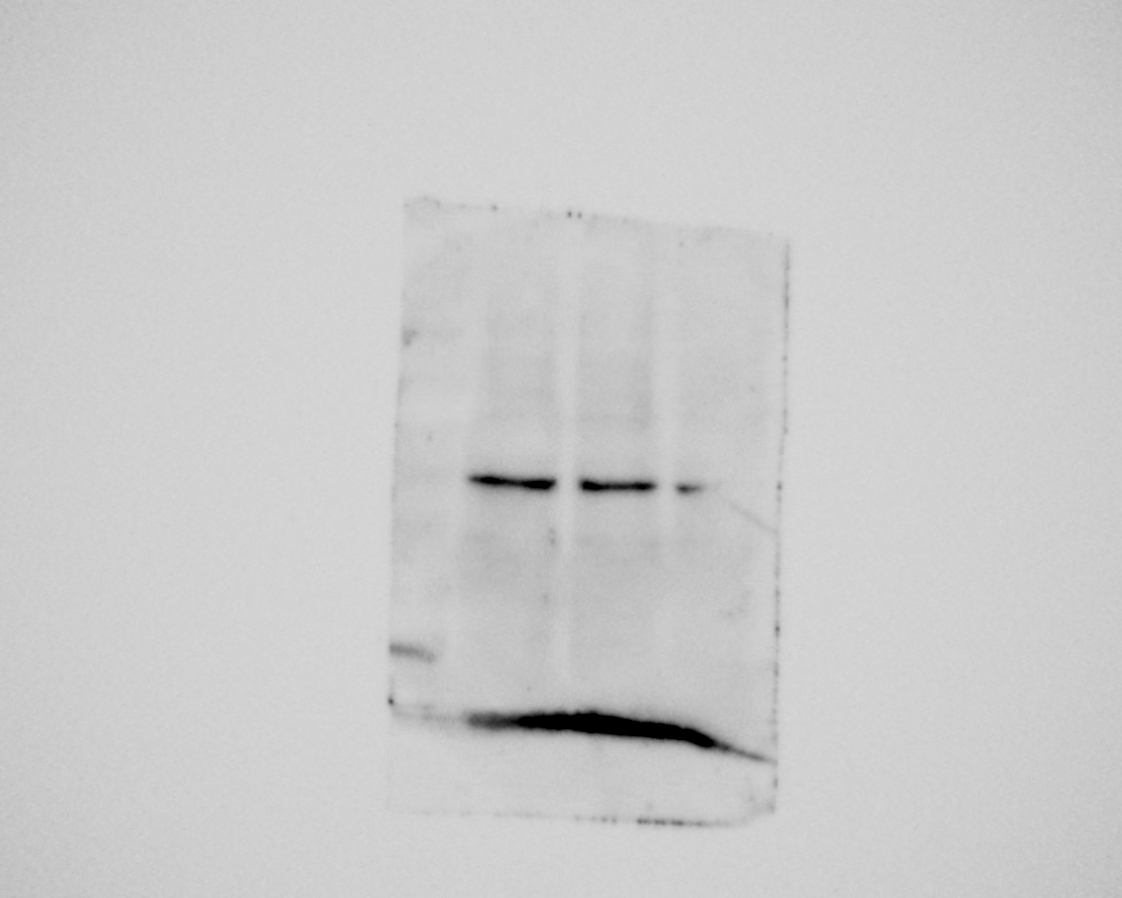

Supplement: Supplementary file 1 — zip [file 12882_2021_2493_MOESM1_ESM.zip › a┬-actin-original-1.jpg]

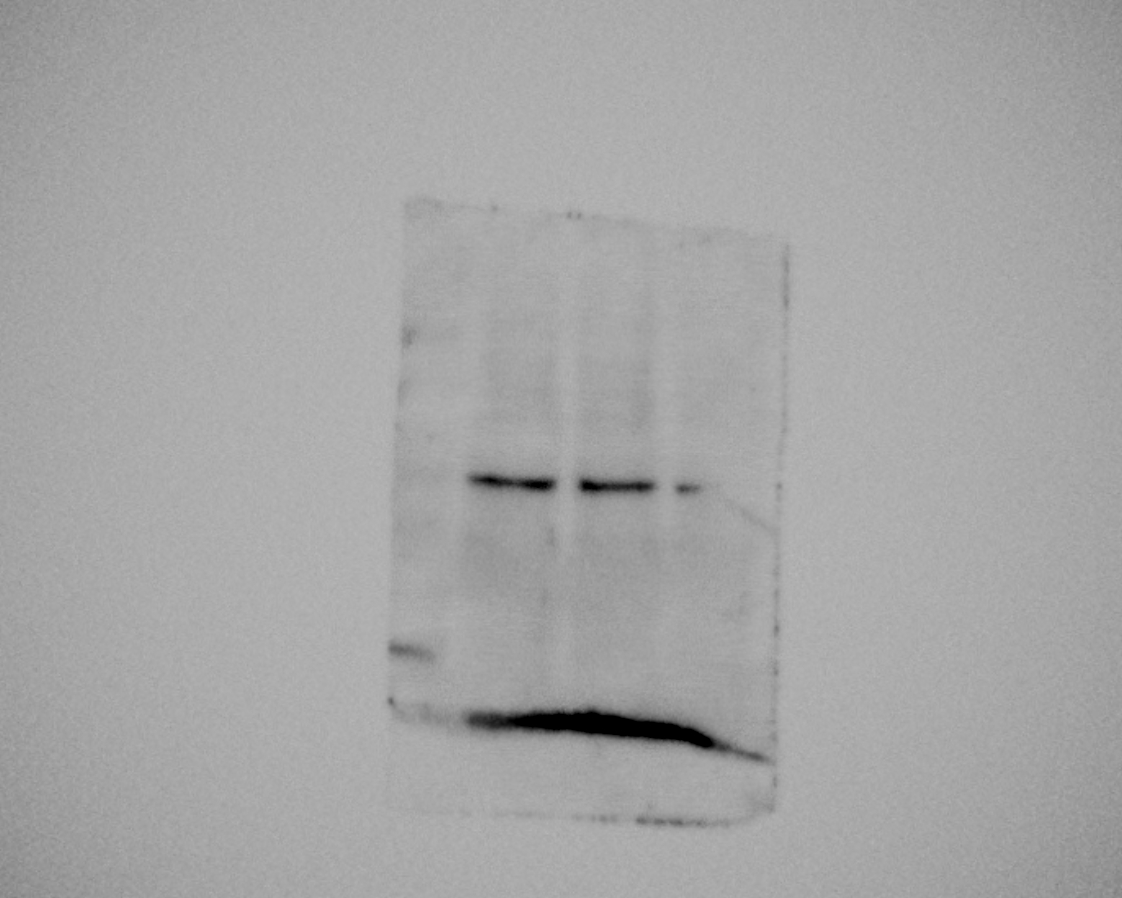

Supplement: Supplementary file 1 — zip [file 12882_2021_2493_MOESM1_ESM.zip › a┬-actin-original-2.jpg]

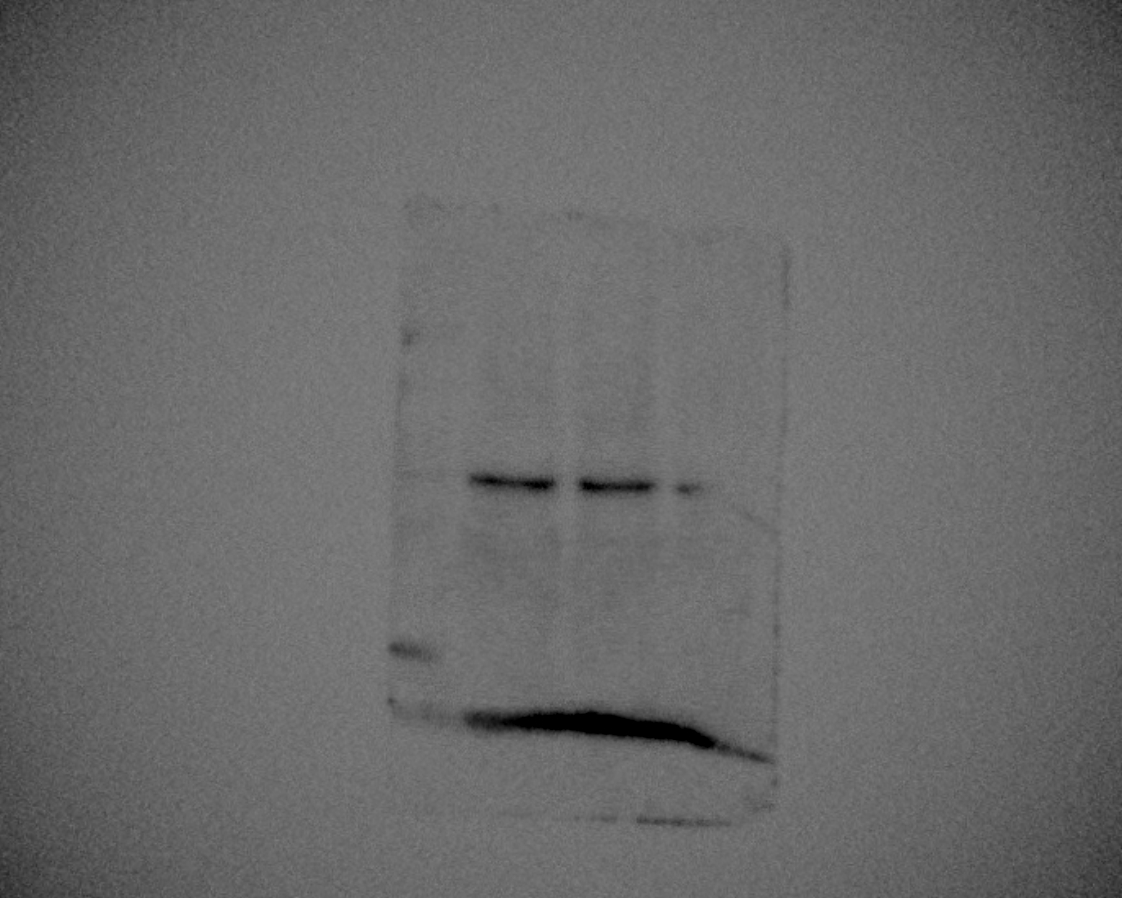

Supplement: Supplementary file 1 — zip [file 12882_2021_2493_MOESM1_ESM.zip › a┬-actin-original-3.jpg]

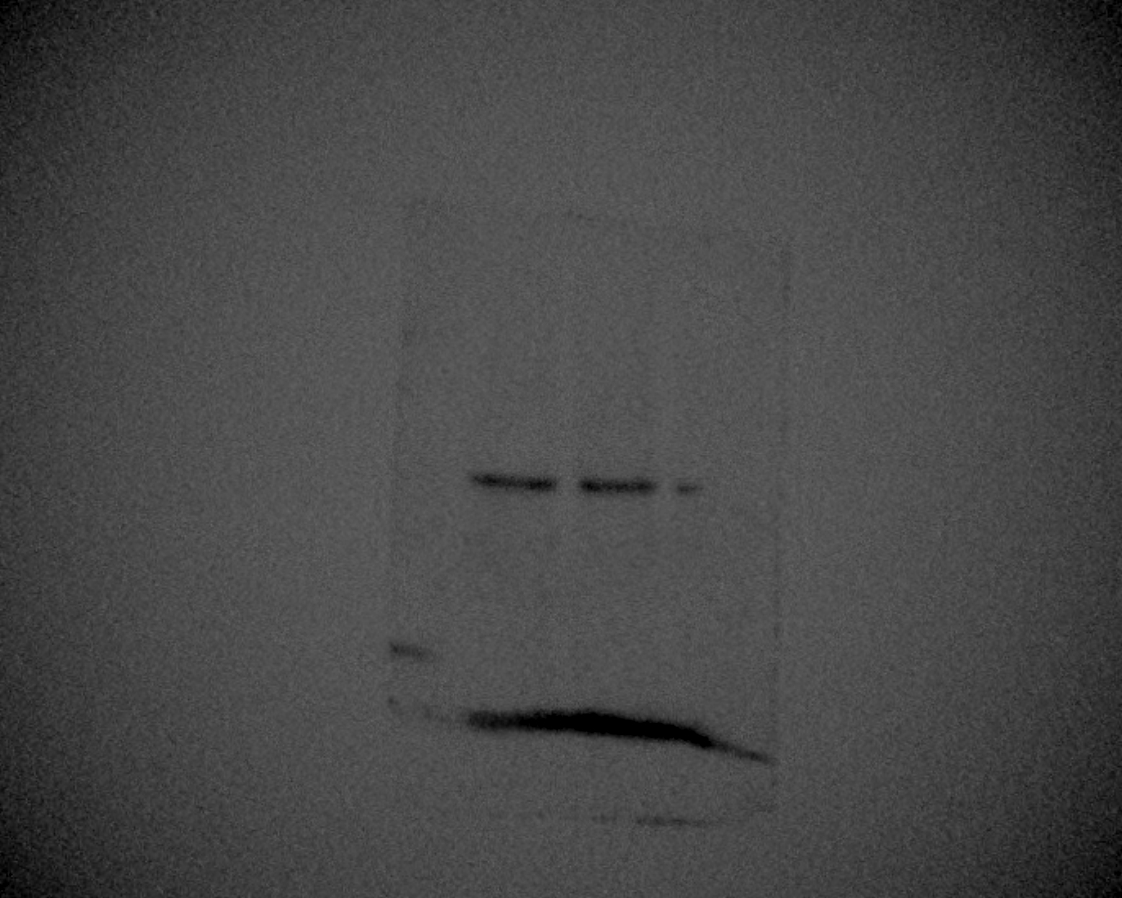

Supplement: Supplementary file 1 — zip [file 12882_2021_2493_MOESM1_ESM.zip › a┬-actin-original-4.jpg]
